# Feed your microbes to deal with stress: a psychobiotic diet impacts microbial stability and perceived stress in a healthy adult population

**DOI:** 10.1038/s41380-022-01817-y

**Published:** 2022-10-27

**Authors:** Kirsten Berding, Thomaz F. S. Bastiaanssen, Gerard M. Moloney, Serena Boscaini, Conall R. Strain, Andrea Anesi, Caitriona Long-Smith, Fulvio Mattivi, Catherine Stanton, Gerard Clarke, Timothy G. Dinan, John F. Cryan

**Affiliations:** 1 APC Microbiome Ireland, Cork, Ireland; 2 Department of Anatomy and Neuroscience, University College Cork, Cork, Ireland; 3 Teagsac Food Research Centre, Moorepark, Fermoy, County Cork, Ireland; 4 Unit of Metabolomics, Research and Innovation Centre, Fondazione Edmund Mach, San Michele all’Adige, Italy; 5 Department of Cellular, Computational and Integrative Biology (CIBIO), University of Trento, Trento, Italy; 6 Department of Psychiatry and Neurobehavioural Sciences, University College Cork, Cork, Ireland

**Keywords:** Psychology, Molecular biology

## Abstract

The impact of diet on the microbiota composition and the role of diet in supporting optimal mental health have received much attention in the last decade. However, whether whole dietary approaches can exert psychobiotic effects is largely understudied. Thus, we investigated the influence of a psychobiotic diet (high in prebiotic and fermented foods) on the microbial profile and function as well as on mental health outcomes in a healthy human population. Forty-five adults were randomized into either a psychobiotic (*n* = 24) or control (*n* = 21) diet for 4 weeks. Fecal microbiota composition and function was characterized using shotgun sequencing. Stress, overall health and diet were assessed using validated questionnaires. Metabolic profiling of plasma, urine and fecal samples was performed. Intervention with a psychobiotic diet resulted in reductions of perceived stress (32% in diet vs. 17% in control group), but not between groups. Similarly, biological marker of stress were not affected. Additionally, higher adherence to the diet resulted in stronger decreases in perceived stress. While the dietary intervention elicited only subtle changes in microbial composition and function, significant changes in the level of 40 specific fecal lipids and urinary tryptophan metabolites were observed. Lastly, microbial volatility was linked to greater changes in perceived stress scores in those on the psychobiotic diet. These results highlight that dietary approaches can be used to reduce perceived stress in a human cohort. Using microbiota-targeted diets to positively modulate gut-brain communication holds possibilities for the reduction of stress and stress-associated disorders, but additional research is warranted to investigate underlying mechanisms, including the role of the microbiota.

## Introduction

With the recognition of the involvement of the gut microbiota in brain processes, mental health and cognitive function, exciting opportunities to produce benefits for brain function by manipulating the microbiota have come into focus. In this regard, the term psychobiotic was coined to describe any exogenous intervention that leads to a bacterially mediated impact on the brain [1, 2]. Thereby, probiotics and prebiotics have shown promising results as psychobiotic agents in both animal and human studies [3, 4]. Furthermore, diet has emerged as a major driver of the composition and function of the microbiota while the influence of habitual dietary patterns on the microbiota has been comprehensively described [5]. At the same time, extensive observational data across many different countries and cultures now link healthy dietary patterns to reduced risk of common mental illnesses [6–8] and emerging trial data suggest that improving dietary habits can improve depressive symptoms [9, 10].

Leveraging this fundamental link between diet and the microbiota, emerging studies are also focusing on the impact of supplementation with single food items (e.g., whole fruits or vegetables or fermented foods), demonstrating some promising results in modulating microbiome-host interactions [11, 12]. While such approaches are important in advancing our understanding of how a specific food impacts human microbiota and health and could lead to the discovery of new functional foods, humans consume a combination of food groups with every meal and studying single foods could overlook the potential synergistic effect dietary components might have, not just on overall health, but also on microbiota diversity and composition [13]. Thus, the study of whole-dietary approaches represents a more realistic path to the development of new dietary psychobiotic interventions. While data from animal models suggest that microbiota-mediated mechanisms (e.g., immune system, hypothalamus-pituitary-adrenal axis, tryptophan metabolism) could underlie this diet–brain connection [14–16], similar studies in human populations are scarce and most clinical studies investigating the effect of diet on anxiety, depression or cognition did not explore microbiota compositional changes [10, 17, 18]. Only, a handful of clinical studies have investigated the efficacy of a microbiota-targeted dietary approach to improve mental health or cognition. For example, one recent study in an obese female population showed that an 8-week nutrition education intervention focused on increasing intake of dietary fiber, vegetable dishes, and milk products shifted the microbiota composition to higher abundance of beneficial microbes (e.g., *Bifidobacterium bifidum*) and decreased depression score [19]. In an elderly population high adherence to the Mediterranean diet over a 12-months period resulted in improvement in global cognition and episodic memory and these improvements were positively associated with key microbial species that responded to the dietary intervention [20].

However, more evidence from human trials is needed to translate pre-clinical findings to a clinical setting and to drive the development of human microbiota-targeted interventions. Therefore, the goal of this study was to investigate the potential of a whole diet psychobiotic approach to modulate the microbiota composition and function, affect responses to and feelings of stress and improve mood in a healthy population.

## Methods

This research was conducted in accordance with the Good Clinical Practice guidelines and the Declaration of Helsinki. The protocol was approved by the Clinical Research Ethics Committee of the Cork Teaching Hospitals (Protocol Number APC102). Informed consent was obtained from all participants prior to enrollment into the study.

### Participants and study design

This study was designed as a single-blind, randomized, controlled study. Healthy adult (male and female) participants with poor dietary habits, aged 18–59 years were recruited from the Cork area between February 2018 and November 2020. The CONSORT flow diagram showing subject enrollment and allocation is depicted in Supplementary Fig. 1. Participants were block randomized (block of 4, stratified by gender) into either intervention (DIET) or control (CONT) group using randomly permuted blocks and were instructed to follow their respective diet for 4 weeks (detailed description of diets below). Other than following the diet, participants were instructed to refrain from introducing any probiotic or other nutritional supplements and to keep physical activity consistent throughout the study.

Participants were not eligible to participate if they had any significant acute or chronic illness, were taking any medication (except for contraceptive pills), were peri-menopausal, menopausal or post-menopausal, were pregnant, or lactating, were not fluent in the English language, were vegan, a habitual daily smoker or had taken any pro-, pre- or antibiotic 4 weeks prior to enrollment in the study. Poor dietary habits were determined by conducting a dietary recall with a trained dietitian. At the initial screening visit, participants were screened for any psychiatric disorder using the MINI International Neuropsychiatric Interview (MINI, Version 7.0.2) and demographic data were collected. Participants also completed the National Adult Reading Test (NART) [21] as a brief measure of verbal IQ as well as the State-Trait Anxiety inventory [22] to measure baseline anxiety levels.

### Dietary intervention

#### Psychobiotic diet

The foods in focus of the psychobiotic diet included those known to influence the microbiota, namely, whole grains, prebiotic fruits and vegetables, fermented foods, and legumes while discouraging consumption of “unhealthy” foods such as sweets, fast food or sugary drinks.

The dietary intervention consisted of one individual, 30-min long education session (baseline) as well as a 15-min long (after 2-weeks) refresher session facilitated by a registered dietitian. During the first session, habitual dietary habits were assessed based on a 7-day food diary the participants completed in the week leading up to the baseline visit. Following this assessment, participants were educated on the components of the study diet, which included consumption of fruits and vegetables high in prebiotic fibers (6–8 servings per day, e.g., onions, leeks, cabbage, apples, bananas, oats), grains (5–8 servings per day) and legumes (3–4 servings per week) as well as fermented foods (2–3 servings per day, e.g., sauerkraut, kefir or Kombucha). For fermented foods, one serving equaled 200 ml or one cup. All other serving sizes were according to the standardized portion size guidance of the Health Service Executive Ireland. Advice including strategies for meal planning, sample menus and recipes that include the specified foods of the study diet as well as on nutrition label reading was given to the participants. Additionally, participants were educated on current Irish food pyramid guidelines and were advised to stay within a 2000–2200 kcals daily intake for females and 2400–2800 kcals for males, which corresponds to the recommended calorie intake per day for moderately active adults according to the Irish dietary guidelines. All education materials (Supplementary Material) were provided to the participants as handouts to take home. Facilitators and barriers to adhering to the study diet were discussed and all remaining questions were answered. In the second session, dietary intake was reviewed again in form of a 7-day food record. Participants were reminded of the components of the diet and were instructed to increase consumption of specific foods if guidelines were not met. Again, all concerns and questions of the participants were discussed. Between study education sessions, participants were encouraged to contact the study dietitian with questions or concerns.

#### Control diet

A dietitian also facilitated the education for the control group and the session was matched in time and dietitian-contact to the diet intervention. A review of dietary habits was completed like the diet group; however, minimal input was provided and focused mainly on the HSE food pyramid. At the 2-week follow up, support was offered to the participants and dietary intake was reviewed using the 7-day food record.

All participants were debriefed after the study and participants in the control arm were provided information about the psychobiotic diet.

### Sample collection

Questionnaire data (as described below) and collection of biological samples was completed pre- and post-intervention.

#### Dietary intake quantification

Dietary intake was quantified using a 7-day food record and food frequency questionnaire (FFQ) that was validated for an Irish adult population [23]. The food diary was completed three times, first in the week leading up to the baseline visit, then in the second week of intervention and finally in the week leading up to the final visit. Instructions on how to complete the food diary (including portion sizes, preparation of food) were provided to participants and the food diary was reviewed by a study dietitian. The FFQ was completed at the pre- and post-intervention visits and asked participants to estimate the intake of specified foods over the previous month. Food groups were derived from the FFQ, by converting each response to a corresponding frequency factor and summing the frequency factors over all food items in a food group to get the average serving per day.

#### Self-report questionnaires

##### Assessment of perceived stress

The Cohen’s perceived stress scale (PSS) [24] was completed by the participants pre- and post-intervention.

##### General health assessment

Gastrointestinal (GI) health was assessed using a GI symptom visual analogue scale. Changes in stool type were captured by the Bristol stool chart [25]. A physical exam (height, weight, blood pressure) was completed at the study visits. Sleep quality was measured using the Pittsburgh Sleep Quality Index (PSQI [26]).

#### Biological samples

##### Sample collection

Plastic containers containing an AnaeroGen sachet (Oxoid AGS AnaeroGen Compact, Fischer Scientific, Dublin) were used to collect freshly voided fecal samples. Participants were instructed to collect the fecal sample as close to the study visit as possible and to keep in a refrigerator until delivery to the laboratory, where it was aliquoted and stored at −80° for later analysis.

Whole blood was collected into 4-ml lithium-heparin-containing tubes (Greiner Bio-One, 454029). Plasma samples were collected into 3-ml K3EDTA tubes (Cruinn Diagnostics Limited, Dublin). Plasma samples were centrifuged at 1500 × *g* for 10 min. The supernatant was aliquoted and stored at −80 °C for later analysis.

Participants were instructed to collect midstream urine samples during the first urination in the morning into a sterile collection tube. Urine samples were stored in the participants refrigerator until delivery to the laboratory. In total, 4 ml of samples were aliquoted into 1 ml of 0.25% sodium azide and stored at −80 °C until further analysis.

#### Fecal microbiota analysis

##### DNA extraction

DNA was extracted from 200 mg of fecal sample using the QIAamp Fast DNA Stool Mini Kit (Qiagen, Germany) combined with repeated bead beating steps, according to methods previously described [27, 28]. DNA concentrations were quantified using Qubit High Sensitivity Kit (Invitrogen). Extracted DNA was then stored at −20 °C until prepared for metagenomic sequencing.

##### Shotgun sequencing library preparation

Whole genome shotgun sequencing library was performed the Nextera XT kit and the library was prepared according to the Nextera XT DNA Library Preparation Guide (Illumina). Briefly, DNA concentrations were diluted to 0.2 ng/μl and fragmented by incubating at 55 °C for 7 min. Paired-end Nextera XT indexes were added and 12 cycles of amplification process were completed. Samples were purified with AMPure XP beads according to the manufacturer’s instructions. In order to calculate molarity, DNA concentration was quantified using the Qubit dsDNA High Sensitivity Assay and amplicon size was measured by Agilent Bioanalyser 2100. Lastly, 1 mM of libraries were pooled before loading on the Illumina NextSeq platform for 150 bp paired-end sequencing.

#### Blood inflammatory profile

Cytokine levels from unstimulated and stimulated blood samples were quantified using the V-PLEX Proinflammatory Panel 1 (human) Kit (MSD, K15049D). Cytokine quantification was done according to the manufacturer’s guidelines with one modification, where 100 μl sample was added directly onto the plate without dilution. C-reactive protein levels were quantified using the kit following the manufacturers guidelines (MSD, K151STD) using a 1000-fold dilution. Sensitivity of each assay is shown in Supplementary Table 1. Specificity of each assay was <0.6%.

##### Fecal metabolomics

Sample analysis was carried out as follows:

##### SCFA

SCFA/metabolite filtrate were acidified using hydrochloride acid, and deuterium labeled internal standards were added. All samples were analyzed in a randomized order. Analysis was performed using a high polarity column (Zebron™ ZB-FFAP, GC Cap. Column 30 m × 0.25 mm × 0.25 μm) installed in a GC (7890B, Agilent) coupled with a quadrupole detector (5977B, Agilent). The system was controlled by ChemStation (Agilent). Raw data were converted to netCDF format using Chemstation (Agilent), before the data were imported and processed in Matlab R2014b (Mathworks, Inc.) using the PARADISe software as previously described [29].

##### Untargeted metabolomics of semi-polar metabolites

The analysis was carried out using a Thermo Scientific Vanquish LC coupled to Thermo Q Exactive HF MS. An electrospray ionization interface was used as ionization source. Analysis was performed in negative and positive ionization mode. The UPLC was performed using a slightly modified version as previously described [30]. Peak areas were extracted using Compound Discoverer 3.1 (Thermo Fisher Scientific). Identification of compounds were performed at four levels; Level 1: identification by retention times (compared against in-house authentic standards), accurate mass (with an accepted deviation of 3 ppm), and MS/MS spectra, Level 2a: identification by retention times (compared against in-house authentic standards), accurate mass (with an accepted deviation of 3 ppm). Level 2b: identification by accurate mass (with an accepted deviation of 3 ppm), and MS/MS spectra, Level 3: identification by accurate mass alone (with an accepted deviation of 3 ppm).

##### Lipidomics

The lipid filtrate were diluted 50 times in eluent mix. Compound extraction was performed in the same way as described for semi-polar metabolites. Data were processed using Compound Discoverer 3.0 (Thermo Fisher Scientific). The retention time of compounds within the same lipid class is estimated using the relation between retention time and the chain length and number of double bonds. Identification of compounds were performed at four levels: Level 1: identification by accurate mass, MSMS spectra and estimated retention time; level 2a is based accurate mass and estimated retention; level 2b is based on accurate mass and MSMS spectra from an external library; level 3: accurate mass and elemental composition alone.

##### Blood and urine targeted metabolomics

Blood and urine metabolomics analysis, targeting key metabolites resulting from human-gut-microbiota co-metabolism of dietary essential amino acids tryptophan, tyrosine, phenylalanine and branched chain amino acids were performed at Fondazione Edmund Mach (Trento, Italy) according to established methods in the laboratory using UHPLC-ESI-MS/MS analysis [31].

##### Cortisol awakening response

Morning saliva samples (the morning of the day of study visit) were collected for assessment of the cortisol awakening response using the Salivette system (Sarstedt, Germany). A total of four samples was collected, with the first one collected upon wakening, the second one 30 min later, and the third and fourth one in 15 min increments. Participants were instructed to not brush their teeth until after all saliva samples were collected, to not eat or drink anything prior to the first sample, and to avoid eating and drinking 15 min prior to the remaining samples. Saliva samples were centrifuged at 1500 × *g* for 5 min, aliquoted, and stored at −80 °C for later analysis.

Salivary cortisol was analyzed in duplicates using the ENZO Life Sciences enzyme-linked immunosorbent assay kits (Exeter, UK) per manufacturer’s instructions. The lower limit of detection was 0.919 nmol/l. Inter- and intra-assay coefficients of variability were 13.4 % and 10.5%.

### Statistical analysis

All data were analyzed using SPSS 25 (IBM, Armonk, NY, USA). Data analysis was performed using the per protocol analysis. Outliers were identified by visual inspection of box blots showing extreme outliers at ±3 standard deviations and consideration was given to removal for extreme outliers. Normality of data was examined using the Shapiro–Wilk statistic and data, if necessary, was transformed using the following transformations: natural log transformation achieved normality for energy, fat, MUFA, cholesterol, magnesium, iron, vitamin A, thiamine, vitamin B6, biotin, folate, fruit, sweets, and vegetables; square root transformation was used to transform omega 6, sodium, manganese, selenium, vitamin D, vitamin C, condiments, sauces and soups, grains, high fat dairy products, other vegetables, processed meat, and refined carbohydrates. PSQI component scores, IPAQ, GI health measures, nutrition outcome variables not listed above, and inflammatory markers were not normally distributed and transformation did not achieve normality.

Differences in outcome measures pre- and post-intervention were analyzed using analysis of covariance and post hoc paired- (within group) or unpaired- (between group) sample two-tailed *t*-test. Homogeneity of variance was checked using Levene’s test of equality of error variances. Age, gender and BMI were included as co-variates for the analysis of study outcome measures. If parametric assumptions were violated and data transformation did not normalize data distribution, the non-parametric Kruskal-Wallis and post hoc paired- (within group) or unpaired- (between group) Wilcoxon singed-rank test (two-tailed) were applied. For cortisol measurements, area under the curve were calculated with respect to ground (AUCg) and increase (AUCi) [32]. Categorical data were assessed using chi-squared test. *p* < 0.05 was considered significant. Data is  expressed as mean ± SEM or median (IQR) unless otherwise indicated.

#### Bioinformatics analysis

We performed quality checks on raw sequences from all fecal samples using FastQC. Shotgun metagenomic sequencing data were then processed through an analysis workflow that utilizes Huttenhower Biobakery pipeline, including Kneaddata, MetaPhlAn3 and HUMAnN3 to obtain species, genes and pathways abundance matrix. Briefly, quality filtering and host genome decontamination (human) was performed using Trimmomatic and Bowtie2 via Kneaddata wrapper program with following parameters: ILLUMINACLIP:/NexteraPE-PE.fa:2:30:10, SLIDINGWINDOW:5:25, MINLEN:60, LEADING:3, TRAILING:3. Taxonomic and functional profiling of the microbial community was performed using MetaPhlan3 and HUMAnN3 using default parameters. Next, gene abundance matrix was further collapsed by Kyoto Encyclopedia of Genes and Genomes (KEGG) Orthology term and Gene Ontology term mapping via “humann_regroup_table” function provided within HUMAnN3.

Further microbiome data-handling was done in R (version 4.1.2) with the Rstudio GUI (version 1.4.1717). The iNEXT library was used to compute alpha diversity for the first three hill numbers (Chao1, Shannon entropy and Simpson Index). Differences in alpha diversity were assessed using linear mixed effect models using the lme4 package. Principal component analysis was performed on centered log-ratio transformed (clr) values as a visual companion to the beta diversity analysis. Zeroes were imputed using the “unif” method. Beta diversity was computed in terms of Aitchison distance (Euclidean distance of clr-transformed counts) and differences in beta diversity were assessed using the PERMANOVA implementation from the vegan library using 10,000 permutations. Microbial volatility was measured as the Aitchison distance between pre- and post-intervention [33].

Gut-brain modules (GBMs) and gut-metabolic modules (GMMs) were calculated using the R version of the Gomixer tool [34]. Differential abundance of taxa and functional modules was assessed by fitting linear mixed effects models on the clr-transformed count tables. To correct for multiple testing (FDR) in tests involving microbiome features, Storey’s *q* value post hoc procedure was performed with a *q* value of 0.2 as a cut off. (Code availability: Custom R scripts are available online at https://github.com/thomazbastiaanssen/Tjazi). Plotting was handled using ggplot2.

#### Metabolomics analysis

Sample analysis was carried out by MS-Omics as follows. Peaks were quantified using area under the curve. Biostatistics were run in R with RStudio GUI. Missing values were imputed by taking 95% of the minimum observed abundance per metabolite. Metabolites that were detected in fewer than 10% of samples were dropped from the analysis. Principal component analysis was performed on CLR-transformed values using the ALDEx2 library. The number of permutations was set to 1000. PERMANOVA followed by a pairwise PERMANOVA was used to find structural differences between treatments on a compositional level. To correct for multiple testing in tests involving metabolomics features, Storey’s *q* value post hoc procedure was performed with a *q* value of 0.2 as a cut off (Code availability: Custom R scripts are available online at https://github.com/thomazbastiaanssen/Tjazi). Metabolites that were found to be altered by diet were mapped to their Human Metabolome Database identifier and subjected to pathway analysis using the MetaboAnalyst online pipeline, choosing the human KEGG library as a reference [35]. Custom Metabolomics figures were generated using ggplot2. Fecal lipids that were significantly altered were subjected to over representation analysis using LIPEA (https://lipea.biotec.tu-dresden.de/home, accessed October 2021) based on a database source including KEGG.

#### Dietary adherence score

A dietary adherence score was calculated based on the ModiMedDiet score as described by [36], with higher scores indicating more adherence to the dietary recommendations. Briefly, a score per serving was assigned to fruit intake (3.33 per serving), vegetables (1.67 per serving), grains, nuts and seeds (2 per serving), legumes (3.33 per serving), and fermented foods (5 per serving). A maximum score of 10 could be achieved for all six categories (fruit; vegetables; grains; legumes, buts and seeds; fermented foods; “unhealthy foods” (e.g., sweets, chocolate, biscuits, fried food) based on the education provided to the participants, summing up to a maximum score of 60. No additional points were given for exceeding the recommended intake. For the unhealthy foods category, 10 points were assigned for 0–3 servings per week, 5 points for 3–6 servings per week and 0 points for ≥6 servings per week.

We performed regression models to predict the changes in outcomes measures based on dietary adherence scores or performed correlation analysis if regression model was deemed inappropriate to explore the relationship between dietary adherence and changes in study outcome measures. Model fit for the regression analysis was determined by checking multicollinearity was checked with VIF being close to 1, residuals were normally and randomly distributed, and no autocorrelation was detected using Durbin Watson statistic of around 2, and influential observations were checked using Cook’s Distance.

## Results

Based on previous microbiota studies from our laboratory [37], a sample size of 30 participants per group was estimated to achieve significant changes in microbiota composition. However, due to the COVID pandemic, recruitment for the study had to be stopped prematurely. Thus, a total of 45 participants (DIET *n* = 24, CONT *n* = 21) completed the study. Two participants dropped out prior to the final visit (time constraints and departure from Ireland due to the COVID-19 lockdown) and were thus not included in the PP analysis.

Baseline demographics are shown in Table 1 and did not differ between groups. The average age was 31.2 (DIET) and 32.2 (CONT) years and more females (~63% in both groups) participated in the study. Most participants (all but 2 in the CONT group) were Caucasian and had a slightly overweight BMI (DIET 28.2 kg/m^2^, CONT 26.8 kg/m^2^). Participants in the DIET group had slightly more years of education (19.3 vs. 17.1, *p* = 0.01) compared to the CONT group; however, predicted intelligent quotient as measured by the NART did not differ between the groups. Body weight did not change over the study period and was not different between groups (data not shown; CONT pre 76 ± 4.1, post 76 ± 4.4; DIET pre 84 ± 3.5, post 82 ± 3.8). Anxiety levels as measured by the STAI were low at baseline and did not differ between the groups (DIET 40 ± 1.7 vs. CONT 28 ± 2, *p* = 0.5).

**Table 1:** Demographic characteristics of study participants.

Variable	Diet (*n* = 24)	CONT (*n* = 21)	*p* value
Age (years)	31.2 ± 1.6	32.2 ± 2.2	0.9
Gender (*n* (%))			0.9
Female	15 (63%)	13 (62%)	
Male	9 (37 %)	8 (38%)	
Mode of delivery (*n*)			0.2
Vaginal	20	14	
Cesarean section	2	6	
Unknown	2	1	
Race (*n*)			0.2
Caucasian	24	19	
Other	0	2	
Years of education	19.3 ± 0.5	17.1 ± 0.7	0.01
NART	110.3 ± 1.44	113.6 ± 1.6	0.14
BMI	28.2 ± 1.1	26.8 ± 1.4	0.4
STAI	40 ± 1.7	38 ± 2	0.5
PSS	15.2 ± 1.1	14.4 ± 1.5	0.3

Data expressed mean ± SEM.

*BMI* body mass index, *NART* National Adult Reading Test, *STAI* State-Trait Anxiety inventory (range 20–80), *PSS* Perceived Stress Scale (range 0–40).

### Diet intervention increased fiber intake and reduced consumption of processed food

Dietary intake pre- and post-intervention is shown in Supplementary Table 2. Importantly, there was a significant group by visit interaction (*F*(1,74) = 4.24, *p* = 0.04) for daily fiber intake (grams/day), which increased in the DIET group to an average consumption of ~29 g/day and was statistically significantly higher in DIET post-intervention compared to CONT (*t*(40) = −4.48, *p* < 0.001). Total calorie intake decreased in both groups (main effect visit (*F*(1,76) = 12.31, *p* = 0.001; CONT *t*(20) = 2.96, *p* = 0.008; DIET *t*(22) = 3.25, *p* = 0.004). Intake of several nutrients changed over the study period in the DIET group and were statistically significantly different between the groups post-intervention, including polyunsaturated fatty acids (*t*(40) = −2.05, *p* = 0.047), omega-3 fatty acids (*Z* = −3.4, *p* = 0.001) as well as some vitamins (vitamin K (*Z* = −2.67, *p* = 0.008, vitamin E (*t*(40) = −2.79, *p* = 0.008) and minerals (calcium (*t*(41) = −2.24, *p* = 0.03, potassium (*t*(42) = −2.52, *p* = 0.02).

Food group analysis derived from the FFQ revealed higher intake of fruit (*t*(43) = −2.81, *p* = 0.007), vegetables (*t*(43) = −2.39, *p* = 0.02) and fermented foods (*Z* = −5.44, *p* < 0.001) in DIET compared to CONT after the intervention. Fermented foods consumed in this cohort included Kefir, Kombucha, yogurt (Activia) and probiotic yogurt drinks (e.g., Actimel, Yakult). Additionally, participants in the DIET group had lower intake of fried foods (*Z* = −2.5, *p* = 0.012) post-intervention compared to participants in the CONT group. Intake of grains (*t*(23) = 3.38, *p* = 0.003) were higher in the DIET group post- compared to pre-intervention, but these were not different between the groups post-intervention. Sugar sweetened beverages (DIET *Z* = −3.29, *p* = 0.001; CONT *Z* = −3.18, *p* = 0.001), processed meats (DIET *t*(23) = −4.49, *p* < 0.001; CONT *t*(20) = −2.31, *p* = 0.032), and refined carbohydrates (DIET *t*(23) = −3.79, *p* = 0.001; CONT *t*(20) = −4.89, *p* < 0.001) consumption was lower in both groups post- compared to pre-intervention, but again did not differ between groups post-intervention.

### Diet intervention reduced perceived stress score

**Fig. 1:**
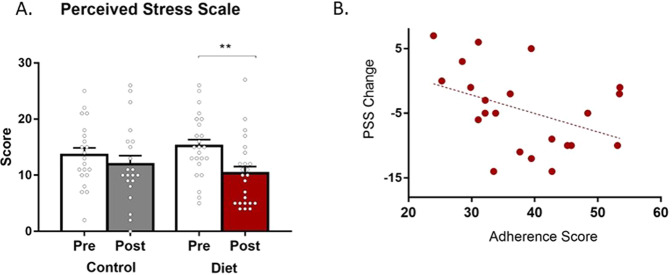
Perceived stress was reduced by dietary intervention and correlated with greater adherence. **A** The diet intervention decreased perceived stress in healthy adult volunteers. Changes in PSS scores in each intervention group are shown. A lower score corresponds to less stress. Data expressed as mean ± SEM; **p ≤ 0.01. **B** Greater adherence to DIET resulted in greater reductions in stress. Adherence to dietary intervention correlated with changes in PSS score. PSS score changes were calculated as absolute number changes between pre- and post-intervention. More negative numbers correspond to greater changes.

There was a main effect of visit on the perceived stress measure (*F*(1,84) = 7.24, *p* = 0.008). Paired within group post hoc analysis revealed a statistically significant 32% decrease in perceived stress in the DIET group (*t*(23) = 3.86, *p* = 0.001), which was not observed in the CONT group (17% decrease) (Fig. 1A).

### Changes in PSS scores are correlated with dietary adherence

Previous nutrition intervention studies have demonstrated that adherence to the diet intervention vary across human cohorts. Similarly, adherence to the dietary intervention in this study population differed between participants. Adherence based on dietary adherence scores varied from 41% (score 25 out of 60) to 100% adherence (score 60 out of 60), with the average being at 65% adherence (score 40 out of 60). Most challenging to adhere to was the consumption of fermented foods which fell below (average 1.7 servings per day) the recommended intake of 2–3 servings per day in the psychobiotic diet intervention group.

Thus, in order to investigate whether higher adherence to the dietary intervention resulted in more pronounced changes in the outcomes of interest in this cohort, dietary adherence scores were calculated and regression analysis with outcome measures was performed. Due to CONT group not receiving the dietary intervention and thus the score not being reflective of diet adherence, this exploratory analysis was only conducted on the DIET group.

Regression analysis (when controlling for age, gender, and BMI) revealed that higher adherence to the study diet resulted in a stronger decrease of PSS score (adj. *R*^2^ = 0.45, std *β* coefficient −0.56, *p* = 0.007), meaning that participants who more closely followed the study diet felt less stressed after the intervention (Fig. 1B). Other outcome measures were not associated with dietary adherence.

### General health questionnaires

In both groups a statistically significant improvement in bowel habit satisfaction (CONT *Z* = −2.9, *p* = 0.003, DIET *Z* = −3.4, *p* = 0.001) and a reduction in interference of bowel habits with life (CONT *Z* = −2.8, *p* = 0.004, DIET *Z* = −3.1, *p* = 0.002) were observed; however, these were not different between the groups post-intervention (Supplementary Table 3). A small number of participants reported current abdominal pain (CONT *n* = 3, DIET *n* = 4) or fullness, bloating or swelling (CONT *n* = 4, DIET *n* = 5) post-intervention; however, these symptoms were mild. Only one participant in the DIET group reported more severe abdominal pain after the intervention. Regarding stool type, a shift toward a normal stool type as measured on the Bristol stool chart (Types 3 and 4) was observed in both groups (CONT 38% pre- vs. 57% post-intervention; DIET 42% pre- vs. 63% post-intervention) (Supplementary Fig. 2). In the CONT group, most participants changed from a soft to a normal stool, whereas in the diet group most participants changed from a hard to a normal stool; however, the change was not statistically significant.

### Biological samples

#### Microbiota composition

The impact of both diets on microbiota composition and function was assessed by whole genome sequencing. Neither microbial α- (Chao 1, Simpson, Shannon) nor β-diversity was impacted by the intervention (data not shown). However, general linear mixed models with Storey’s *q* value post hoc correction revealed a statistically significant increase in *Blautia wexlerae* (*q* = 0.11) and decrease in *Blautia obeum* (*q* = 0.14), *Coprococcus comes* (*q* = 0.11), *Dorea longicatena* (*q* = 0.11), *Eubacterium rectale* (*q* = 0.11), *Gemmiger formicilis* (*q* = 0.14), and *Bifidobacterium longum* (*q* = 0.16) in the DIET group post- compared to pre-intervention.

**Fig. 2:**
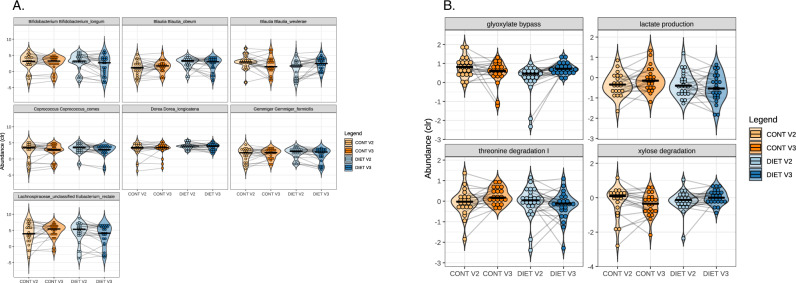
Microbiota composition and function that were affected by dietary intervention. **A** Relative abundance of microbial taxa differentially affected by dietary treatment. V2 refers to pre-intervention abundance, V3 to post-intervention; *n* (CONT V2) = 21; *n* (CONT V3) = 21; *n* (DIET V2) = 24; *n* (DIET V3) = 24). **B** Changes in microbial functions in DIET and CONT group throughout dietary intervention. V2 refers to pre-intervention levels, V3 to post-intervention; *n* (CONT V2) = 21; *n* (CONT V3) = 21; *n* (DIET V2) = 24; *n* (DIET V3) = 24).

When adjusting for co-variates, results did not change, suggesting that effect of diet observed in these seven species of interest is invariant to age, gender, and BMI. Violin boxplots of the relative abundance of microbial species that differed between intervention groups are shown in Fig. 2A.

#### Functional analysis

To investigate functional changes in the microbiota in response to dietary intervention, GBM and GMM were analyzed according to similar general linear mixed models as for differential species abundance. No effect on GBM was observed. In the GMM, subtle upregulation of the glyoxylate bypass (*q* = 0.09), and xylose degradation (*q* = 0.15) and downregulation of lactate production (*q* = 0.15) and threonine degradation I (*q* = 0.15) was observed in the DIET group (Fig. 2B). Again, when adjusting for co-variates, these results did not change, suggesting that the effect of diet observed were invariant to age, gender, and BMI.

### Changes in PSS scores are correlated with microbial volatility

**Fig. 3:**
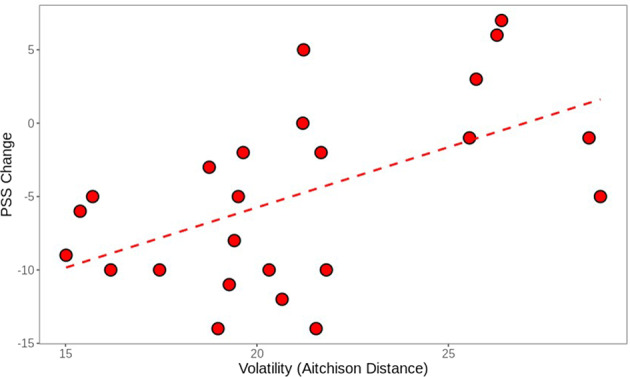
Volatility is associated with greater changes in PSS scores in dietary intervention group. Lower volatility (more stable microbiota) correlated with greater changes in PSS score. PSS score changes were calculated as absolute number changes between pre- and post-intervention. More negative numbers correspond to greater changes.

In order to investigate whether overall microbial volatility (changes in microbial diversity pre- and post-intervention) was related to specific changes in outcomes of interest, the association between volatility and outcome measures was investigated. A positive correlation between volatility and changes in PSS (*r* = 0.53, *p* = 0.007, Fig. 3) was observed, meaning that participants with less volatility had greater decrease in PSS. This correlation was not observed in the CONT group (*r* = 0.14, *p* = 0.53).

Surprisingly, volatility was not linked to dietary intake, suggesting that the diet did not impact microbial stability. Other outcome measures were not statistically significant in regression analysis after accounting for multiple comparisons. Similarly, microbial taxa or function did not statistically significantly correlate with adherence score.

### Fecal metabolomics

**Fig. 4:**
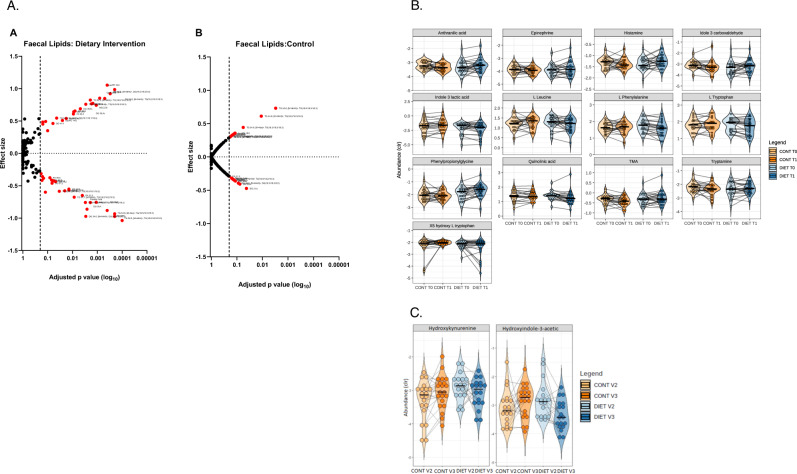
Alterations in fecal, urine and plasma metabolites. **A** Volcano plot showing fecal lipid metabolites statistically significantly altered by dietary intervention (A) and in control group (B). **B** Changes in urinary metabolites after dietary intervention. Only metabolites that changed statistically significantly are shown. V2 refers to pre-intervention levels, V3 to post-intervention; *n* (CONT V2) = 21; *n* (CONT V3) = 21; *n* (DIET V2) = 24; *n* (DIET V3) = 24). **C** Changes in plasma metabolites after dietary intervention. 5-hydroxyindole-3-acetic acid and 3-hydroxykynurenine statistically significantly decreased in the diet group; V2 refers to pre-intervention levels, V3 to post-intervention; *n* (CONT V2) = 21; *n* (CONT V3) = 21; *n* (DIET V2) = 24; *n* (DIET V3) = 24).

A comprehensive panel of metabolites in fecal samples was screened to investigate changes induced by the dietary intervention. A total of 40 lipid metabolites were statistically significantly affected within the DIET group, which was not observed in the CONT group (Fig. 4A). Other metabolites (SCFA, semi-polar metabolites) were not affected by the intervention.

### Plasma and urine metabolomics

In order to investigate potential peripheral changes elicited by the diet intervention and identify underlying microbial mechanisms, plasma and urine metabolites were analyzed. A total of 50 metabolites were detected in urine samples, of which 13 were statistically significantly affected in the DIET group (Fig. 4B). Specifically within the DIET group, a decrease in quinolinic acid, L-tryptophan, and L-phenylalanine, was observed, while phenylpropionylglycine increased after DIET intervention. 34 metabolites were quantified in the plasma of which 2 were statistically significantly affected within the DIET group, namely 5-hydroxyindole-3-acetic acid (*q* = 0.11) and 3-hydroxykynurenine (*q* = 0.2) (Fig. 4C).

### Sleep quality and other biological measures

There was a main effect of visit (*F*(1,84) = 12.58, *p* = 0.001) on sleep quality. Post hoc paired sample *t*-test revealed that overall sleep improved (shown by lower scores on PSQI) in both groups (CONT *t*(20) = 2.69, *p* = 0.01, DIET *t*(23) = 4.03, *p* = 0.001) (Supplementary Fig. 3a). Investigating the components of the PSQI revealed that this difference could have been mainly driven by the improved subjective sleep quality (component 1), which was statistically significantly improved in the DIET group compared to the CONT group (*Z* = −2.73, *p* = 0.02) (Supplementary Fig. 3b). Sleep duration (component 3) statistically significantly improved in CONT (*Z* = −2.31, *p* = 0.02), but the score was not different between groups post-intervention.

In order to investigate the impact of the dietary intervention on potential pathways of the gut-brain communication, immune markers and cortisol levels (as marker for the hypothalamus-pituitary-adrenal axis) were investigated. However, neither cortisol awakening response (Supplementary Fig. 4) nor measured immune markers (Supplementary Table 4) were affected by dietary intervention.

## Discussion

Given that the gut microbiota has emerged as a key conduit for brain processes, mood and behavior, developing microbiota-targeted approaches that can benefit mental health and brain function is an exciting opportunity, not only for improving the mental health of populations but also in the quest of developing new therapeutic approaches for patients who might be resistant to traditional therapies. Although the more traditional psychobiotic candidates, such as probiotic and prebiotic supplementation, are promising approaches, using whole dietary approaches offers a number of advantages. This includes the benefits associated with nutritional needs met primarily through whole diet, a more accessible way due to the necessity to consume a food daily, costs of supplemental products as well as the potential synergistic effects in the microbiota that could be elicited by complex diets. While some trials have shown promise is using a microbiota-targeted approach [19, 20], it is unknown if such an approach could reduce stress in a healthy adult population. Thus, the aim of this study was to explore how a dietary intervention rich in foods known to benefit the microbiota composition could influence human microbiota, stress, and peripheral markers of health.

Most notably we observed a reduction in perceived stress in the cohort undergoing the dietary intervention, although there was no difference between the groups post-intervention. Importantly, reduction in perceived stress was dose dependent, meaning that higher adherence to the study diet resulted in stronger decreases in the PSS score. Strong evidence between stress and health behaviors, including healthy diet quality, has been presented in cross-sectional studies [38, 39]. Regarding interventional studies, lifestyle approaches, some in combination with a dietary component [40, 41], and single nutrient or food supplementation [42] demonstrated reductions in perceived stress in participants, and dietary interventions have successfully reduced symptoms of depression [10], even in nonclinical depression [9]; however, less is known about the impact of whole dietary interventions on perceived stress and the limited studies availavle reported inconclusive results [43]. Although improvements in stress through microbiota-targeted interventions (i.e., probiotics, fermented beverage) have previously been observed [44–46], our findings are one of the first to provide key evidence for the use of whole diet approaches to alleviate stress perception in human cohorts. Additionally, a link between low volatility, a measure of change in microbiota composition between timepoints, and greater reductions in perceived stress were observed. Intriguingly, this observation corresponds to previous reported results from our laboratory, linking greater volatility to greater perceived stress [47] and literature also shows that a diverse diet leads to a stable microbiota [48]. Altogether, future studies should investigate the hypothesis that the psychobiotic diet leads to a more stable microbiota which resulted in greater changes in perceived stress.

Contrary to our hypothesis, we only observed subtle changes in microbial composition and function. While this lack in microbial changes could partly be attributed to other study factors, such as sample size or short duration of the study (which might not have allowed enough time for new taxa to emerge and microbial remodeling to be elicited), these observations are in line with earlier studies suggesting difficulty in observing global microbial changes in response to dietary interventions, such as increased dietary fiber intake [49], indicating that habitual diet might have a stronger impact on the gut microbiota [50]. Limited effect on diversity and minimal microbial changes in response to whole, plant based food diets [51] have previously been reported and the effect might be larger in animal than human studies due to the interindividual heterogeneity of the human microbiota [48]. Likewise, the amount of fermented foods consumed by the study participants was below the recommended intake, it could have been insufficient to elicit broad microbial changes as observed in previous studies [49]. To date, specific recommendations on consumption of fermented foods are generally absent in dietary guidelines around the world and there is no consensus on the amount that should be consumed to elicit a health benefit [52]. In available studies, the amount as well as the type of fermented food and duration of consumption studied is also highly variable. For example, in a observational study analyzing samples from 115 individuals in the American Gut Project, consumption of fermented foods 1 or 2 times per week or once per day had a dose dependent effect on gut community measured by β-diversity and abundance of bacterial taxa (e.g., *Bacteroides*, *Pseudomonas*, *Dorea*, *Prevotella*, *Oscillospira*, *F. prausnitzii*, *Lactobacillus* spp.) as well as microbial functional profile [53]. On the other hand, in a recent intervention study, consumption of 6 servings per day of various fermented foods elicited changes in the microbial diversity and immune system [49] and in another recent study, daily consumption of 8 oz of a fermented dairy beverage increased *Lactobacillus* abundance and resulted in improvements in relational memory [12]. Similarly, a high fiber intervention of 40–50 grams of fiber per day (which is 10–15 grams higher than the fiber consumed in this study and than the recommended intake in various countries) specifically increased fiber degrading bacteria such as *Bifidobacterium* or *Lactobacillus* [54]. Thus, in short intervention studies a higher amount a fiber is perhaps needed to elicit microbial changes. Although we did not find changes on the compositional or functional level of the microbiota, an impact on fecal metabolites, specifically lipids, was observed. While these changes could be attributed to the reduced intake of high fatty foods by the participants, investigations have also revealed that several probiotic microbes can induce changes in lipid metabolites [55, 56], which could have consequences for peripheral health. Thus, further investigations into the importance of changes in lipid metabolism and how these changes could related to mental health are warranted.

Different mechanisms have been proposed to be underlying the microbiota-gut-brain communication, including the immune system, microbial metabolites or the hypothalamus-pituitary adrenal (HPA) axis. Although causational relationships cannot be established from human cohorts, it is important to investigate potential biological changes that could be associated with mental health benefits observed. Such results could then inform future animal studies to investigate mechanistic pathways. Here, it appears that neither the immune system nor the HPA axis were underlying the stress-relieving effect of the psychobiotic diet. While the main components of the psychobiotic diet, namely dietary fiber and fermented foods, have been associated with anti-inflammatory effects [57–60] and a recent study demonstrated that a diet rich in fermented foods could improve inflammatory status of the host [49], the lack of changes in the immune profile could be due to our limited sample size, short duration of the intervention and the amount of fermented food consumed by participants in the present study. Additionally, besides having poor dietary habits at baseline, the population was healthy so that changes in a population without an inflammatory state (average CRP levels were <10 mg/l, which is the standard for a normal CRP test) at baseline might not be detectable. Other aspects of the immune system that are more prone to dietary modulation in a healthy population (such as white blood cell profile [61] or other monocytes, CD8+ or CD4+ T-cells [49]) or more comprehensive profiling could give more insight into the immunomodulatory effect of fermented foods.

Another potential pathway of the microbiota-brain communication are microbial metabolites. Here, intriguingly, urine metabolomics revealed that urine metabolites were altered in the tryptophan metabolism. Tryptophan metabolism has been shown to be closely regulated by the microbiota and can serve as important bioactive messengers in the microbiota-brain communication [62]. Additionally, tryptophan metabolism has been suggested to be involved in many brain related disorders [63] and previous studies have shown that the metabolites can be modulated by a Mediterranean diet [64]. Here, it appeared that the dietary intervention reduced the kynurenine pathway metabolite, quinolinic acid, which is a NMDA agonist and can exert neurotoxic effects which can disrupt neurotransmission in high concentrations [65, 66]. Additionally, we observed alterations in fecal lipid metabolites, which could be attributed to the observed dietary changes that included decrease in total fat and increase in monounsaturated fat consumption. On the other hand, recent studies have reported potential associations between changes in lipid metabolites and depression-like behavior in mice [67] as well as post-stroke depression [68], suggesting that the microbiota might influence mood through regulation of lipid metabolism. While these observations allow us to formulate exciting hypothesis, the physiological significance of these findings remains to be determined, as we could not associate the urine or fecal metabolites with changes in PSS.

Although, to the best of our knowledge, this study is one of the first investigating a psychobiotic diet approach to improve mental well-being, we acknowledge its limitations in interpreting the findings. Firstly, the sample size, restricted due to the COVID-19 pandemic, the University closure and related national restrictions, limits the generalization of the study findings. Secondly, the short duration of the study could have limited the biological and microbial changes observed and the possibility of more pronounced changes during longer interventions needs to be considered. Thirdly, the intervention included a nutrition education without assessment of behavioral change and while food intake was assessed with comprehensive diet record, these methods are susceptible to measurement error and bias in estimating food intake. Despite the observed increase in dietary fiber intake, dietary adherence revealed a variation in how well participants were able to follow the intervention. Furthermore, while a best effort was put forth to assure blinding of the participants to the study diets, participants could have guessed their group allocation due to the nature of the nutrition education, which may have affected their responses. Lastly, the intervention included a cohort of healthy individuals, potentially limiting some of the changes induced by the diet, specifically the biological changes, as a change in an already healthy individual would be minimal. Future studies in clinical cohorts are warranted to investigate potential therapeutic use of the study diet.

## Conclusion

In conclusion, a short term psychobiotic dietary intervention improved perceived stress in a healthy population, while eliciting specific metabolic changes in the gut microbiota. However, results should be interpreted with caution as, although improvements in stress responses were only observed within the intervention group, there were no significant differences between groups after the intervention. Nevertheless, given the fundamental influence of stress on the risk for developing other chronic diseases such as depression, these findings hold promise as potential therapeutic and preventative approaches. Thereby, using dietary and lifestyle approaches to address mental health concerns will become increasingly important and could become reflected in future dietary guidelines. Although our study provides one of the first data in the interaction between diet, microbiota and mental health, the subtle changes observed in biological outcomes and limited sample size highlights the necessity for future larger and longer duration studies to confirm the stress-alleviating effect of the psychobiotic diet. Additionally, aspects of other diets known to benefit mental health such as the MIND Diet, ketogenic diet or intermittent fasting need to be considered. Lastly, underlying microbial influences need to be investigated and future pre-clinical experiments are required to explore causality and decipher mechanistic pathways.

## Supplementary information

Supplementary Tables and Figures

Diet Education Material

## Data Availability

Whole genome sequences are available at: European Nucleotide Archive, Accession ID PRJEB56264.
