# Supplementary material for: Feed your microbes to deal with stress: a psychobiotic diet impacts microbial stability and perceived stress in a healthy adult population
Source: Mol Psychiatry. 2022 Oct 27;28(2):601–10. doi: 10.1038/s41380-022-01817-y (PMC9908549; doi:10.1038/s41380-022-01817-y)
Supplement: Supplementary file 1 — Supplementary Tables and Figures [file 41380_2022_1817_MOESM1_ESM.docx]

**Supplemental Tables and Figures**

**Supplementary Table 1**. Sensitivity of blood inflammatory assays.

|  | **LLOQ (pg/mL)** | **LLOD Range (pg/mL)** |
| --- | --- | --- |
| **IL-10** | 0.30 | 0.02-0.08 |
| **IL-8** | 0.59 | 0.03-0.14 |
| **IFN-γ** | 1.76 | 0.21-0.62 |
| **IL-12 p70** | 1.22 | 0.02-0.89 |
| **IL-6** | 0.63 | 0.05-0.09 |
| **TNF-α** | 0.69 | 0.01-0.13 |
| **CRP** | 27.60 | 0.69-19.8 |
| LLOQ = Lowest Limit of Quantification | | |
| LLOD = Lowest Limit of Detection | | |

**Supplementary Table 2.** Summary of nutrient and food group intake

| **Nutrient** | **Control** | | **Diet** | |  |
| --- | --- | --- | --- | --- | --- |
|  | **Pre-intervention** | **Post-Intervention** | **Pre-intervention** | **Post-Intervention** | **p-value^1^** |
| Energy (Kcal) | 1802 ± 112 | 1475 ± 91** | 2030 ± 114 | 1649 ± 101** | 0.19 |
| Carbohydrate (g) | 177 ± 11 | 159 ± 11 | 209 ± 12 | 177 ± 11* | 0.16 |
| Protein (g) | 79 ± 5.8 | 75 ± 5.9 | 78 ± 4.9 | 79 ± 5.3 | 0.30 |
| Fat (g) | 78 ± 5.5 | 58 ± 4.2** | 90 ± 5.2 | 67 ± 5.8** | 0.26 |
| Fibre (g) | 14 ± 1.2 | 17 ± 1.4 | 17 ± 1.2 | 29 ± 1.9*** | <0.001 |
| Starch (g) | 113 ± 7.5 | 95 ± 7.4* | 128 ± 7.0 | 96 ± 6.7** | 0.85 |
| Saturated Fat (g) | 29 ± 2.1 | 22 ± 1.6** | 36 ± 1.9 | 23 ± 1.9*** | 0.52 |
| Trans-fatty acid (g) | 0.83 ± 0.08 | 0.67 ± 0.07 | 0.89 ± 0.06 | 0.69 ± 0.06* | 0.94 |
| Cholesterol (mg) | 302 ± 39 | 287 ± 33 | 199 ± 20 | 192 ± 26 | 0.006 |
| Monounsaturated fat (g) | 25 ± 2.3 | 18 ± 1.5** | 26 ± 2.0 | 22 ± 2.4* | 0.9 |
| Polyunsaturated fat (g) | 9.7 ± 1.01 | 8.1 ± 0.8 | 10 ± 0.97 | 11 ± 1.2 | 0.047 |
| Omega-6 FA (g) | 4.6 ± 0.7 | 4 ± 0.51 | 5.1 ± 0.7 | 6.4 ± 0.9 | 0.09 |
| Omega-3 FA (g) | 0.9 ± 0.13 | 0.9 ± 0.15 | 0.9 ± 0.12 | 1.8 ± 0.17** | 0.001 |
| ***Minerals*** | | | | |  |
| Sodium (mg) | 2140 ± 170 | 1922 ± 175 | 2454 ± 157 | 1917 ± 18* | 0.93 |
| Potassium (mg) | 2109 ± 156 | 2253 ± 150 | 2231 ± 148 | 2842 ± 176*** | 0.02 |
| Chloride (mg) | 3186 ± 251 | 2877 ± 258 | 3610 ± 215 | 3056 ± 254 | 0.73 |
| Calcium (mg) | 622 ± 56 | 602 ± 46 | 713 ± 47 | 773 ± 51 | 0.03 |
| Phosphorus (mg) | 1049 ± 79 | 1084 ± 76 | 1040 ± 75 | 1262 ± 84 | 0.13 |
| Iron (mg) | 8.12 ± 0.62 | 8.76 ± 0.76 | 8.74 ± 0.59 | 11.34 ± 0.91** | 0.05 |
| Copper (mg) | 0.80 ± 0.06 | 0.85 ± 0.05 | 0.85 ± 0.06 | 1.14 ± 0.07*** | 0.03 |
| Zinc (mg) | 7.79 ± 0.74 | 7.28 ± 0.57 | 7.15 ± 0.53 | 8.84 ± 0.59 | 0.11 |
| Manganese (mg) | 2.24 ± 0.22 | 2.93 ± 0.26 | 2.45 ± 1.1 | 3.99 ± 0.3*** | 0.008 |
| Selenium (µg) | 39.9 ± 3.66 | 41.02 ± 3.8 | 33.01 ± 3.09 | 38.8 ± 3.55 | 0.68 |
| Iodine (µg) | 96.9 ± 10.22 | 96 ± 9.4 | 92.2 ± 9.46 | 117 ± 8.5 | 0.10 |
| ***Vitamins*** | | | | |  |
| Vitamin A (µg) | 540 ± 64 | 675 ± 108 | 587 ± 79 | 873 ± 80*** | 0.09 |
| Vitamin D (µg) | 2.33 ± 0.27 | 2.54 ± 0.3 | 1.75 ± 0.24 | 2.67 ± 0.29* | 0.66 |
| Vitamin E (mg) | 6.99 ± 0.54 | 5.65 ± 0.50 | 6.66± 0.51 | 8.1 ± 0.69 | 0.008 |
| Vitamin K (µg) | 24.7 ± 5.7 | 46.3 ± 5.6*** | 27.6 ± 5.5 | 68.1 ± 5.6*** | 0.008 |
| Thiamine (B1) (mg) | 1.08 ± 0.07 | 1.33 ± 0.13 | 1.28 ± 0.1 | 1.67 ± 0.12** | 0.02 |
| Riboflavin (B2) (mg) | 1.22 ± 0.09 | 1.27 ± 0.11 | 1.25 ± 0.11 | 1.38 ± 0.1 | 0.44 |
| Niacin (B3) (mg) | 32.3 ± 2.9 | 32.7 ± 2.9 | 31.7 ± 2.2 | 31.5 ± 2.5 | 0.74 |
| Pantothenic Acid (B5) (mg) | 4.42 ± 0.4 | 4.46 ± 0.26 | 4.25 ± 0.37 | 4.88 ± 0.31 | 0.75 |
| Vitamin B6 (mg) | 1.43 ± 0.14 | 1.48 ± 0.12 | 1.43 ± 0.14 | 1.8 ± 0.12** | 0.061 |
| Biotin (B7) (µg) | 28.9 ± 2.32 | 28.63 ± 1.78 | 23.81 ± 2.31 | 31.79 ± 2.12** | 0.26 |
| Folates (B9) (µg) | 311 ± 34.7 | 336 ± 28.01 | 336 ± 27.7 | 449 ± 42.8** | 0.054 |
| Vitamin B 12 (µg) | 4.53 ± 0.58 | 4.19 ± 0.34 | 3.8 ± 0.4 | 4.04 ± 0.32 | 0.97 |
| Vitamin C (mg) | 47.3 ± 9.1 | 61.3 ± 6.5 | 52.3 ± 8.1 | 101 ± 10.1*** | 0.002 |
| **Food group** |  |  |  |  |  |
| Fruit | 0.9 ± 0.2 | 1.9 ± 0.5** | 1.6 ± 0.3 | 3.2 ± 0.5*** | 0.007 |
| Vegetables (total) | 2.4 ± 0.4 | 3.2 ± 0.5 | 3 ± 0.4 | 5.4 ± 0.7* | 0.02 |
| Cruciferous vegetables | 0.3 ± 0.1 | 0.5 ± 0.1* | 0.3 ± 0.1 | 1 ± 0.2*** | 0.054 |
| Green and orange vegetables | 0.7 ± 0.1 | 0.9 ± 0.2 | 0.6 ± 0.1 | 1.6 ± 0.3*** | 0.031 |
| Starchy vegetables | 0.2 ± 0.1 | 0.4 ± 0.1 | 0.3 ± 0.1 | 0.4 ± 0.1 | 0.54 |
| Other vegetables | 1.2 ± 0.2 | 1.4 ± 0.2 | 1.5 ± 0.2 | 2.5 ± 0.3*** | 0.006 |
| Potatoes | 0.4 ± 0.1 | 0.3 ± 0.1 | 0.5 ± 0.1 | 0.3 ± 0.1 | 0.94 |
| Vegetarian protein foods | 0.6 ± 0.2 | 0.5 ± 0.1 | 0.6 ± 0.2 | 0.5 ± 0.1 | 0.27 |
| Fish | 0.2 ± 0 | 0.2 ± 0 | 0.2 ± 0 | 0.2 ± 0 | 0.85 |
| Poultry | 0.3 ± 0 | 0.3 ± 0.1 | 0.3 ± 0 | 0.3 ± 0 | 0.79 |
| Processed meat | 0.5 ± 0.1 | 0.4 ± 0.1* | 0.6 ± 0.1 | 0.3 ± 0.1** | 0.17 |
| Red meat | 0.4 ± 0.1 | 0.4 ± 0.1 | 0.5 ± 0.1 | 0.4 ± 0.1 | 0.77 |
| Legumes, nuts, and seeds | 0.1 ± 0 | 0.1 ± 0 | 0.2 ± 0 | 0.3 ± 0.1 | 0.48 |
| Grains | 1.6 ± 0.4 | 1.5 ± 0.3 | 1.2 ± 0.2 | 2.3 ± 0.4** | 0.07 |
| Cereals | 0.3 ± 0.1 | 0.3 ± 0.1 | 0.3 ± 0.1 | 0.4 ± 0.1 | 1.0 |
| Refined carbohydrates | 1.5 ± 0.2 | 0.7 ± 0.1*** | 1.5 ± 0.2 | 0.6 ± 0.1*** | 0.71 |
| Fried foods | 0.6 ± 0.1 | 0.4 ± 0.1 | 0.5 ± 0.1 | 0.2 ± 0** | 0.012 |
| High fat dairy products | 2.1 ± 0.3 | 1.3 ± 0.2* | 1.7 ± 0.3 | 1.4 ± 0.2 | 0.86 |
| Low fat dairy products | 0.5 ± 0.2 | 0.5 ± 0.1 | 0.3 ± 0.1 | 0.6 ± 0.1 | 0.57 |
| Sweets | 4.8 ± 1 | 1.2 ± 0.2*** | 3.9 ± 0.6 | 1 ± 0.2*** | 0.61 |
| Snacks | 0.7 ± 0.1 | 0.3 ± 0.1 | 0.7 ± 0.1 | 0.5 ± 0.1 | 0.03 |
| Coffee | 1.4 ± 0.4 | 1.1 ± 0.3 | 1.4 ± 0.3 | 1.2 ± 0.3 | 0.92 |
| Sweetened, sugary beverages | 1 ± 0.3 | 0.4 ± 0.1** | 1.4 ± 0.3 | 0.5 ± 0.2** | 0.68 |
| Alcoholic beverages | 0.4 ± 0.1 | 0.3 ± 0.1 | 0.3 ± 0.1 | 0.2 ± 0.1 | 0.5 |
| Non-dairy beverages | 1.8 ± 0.4 | 1.2 ± 0.3 | 1 ± 0.2 | 0.8 ± 0.2 | 0.13 |
| Casseroles and mixed dishes | 0.1 ± 0 | 0.1 ± 0 | 0.1 ± 0 | 0 ± 0 | 0.32 |
| Condiments, sauces, and soups | 1.4 ± 0.2 | 1 ± 0.2 | 1.5 ± 0.2 | 1.4 ± 0.2 | 0.16 |
| Fermented foods | 0 ± 0 | 0 ± 0 | 0 ± 0 | 1.7 ± 0.2*** | <0.001 |

^1^p-value for post-intervention diet vs. control; within same row and group difference between pre-and post-intervention at *p≤0.05, **p≤0.01, ***p≤0.001; data shown as mean±SEM

FA – fatty acids

**Supplementary Table 3**. Gastrointestinal satisfaction improved in both groups

|  | **CONT** | | **DIET** | |  |
| --- | --- | --- | --- | --- | --- |
| **Variable** | **Pre-intervention** | **Post-intervention** | **Pre-intervention** | **Post-intervention** | **p-value** |
| Current abdominal pain  Yes (%) (severity^2^)  No (%) | 2 (9.5%)  (16.7 ± 0.09)  19 (90.5%) | 3 (14%)  (11 ±0.07)  18 (86%) | 4 (17%)  (15 ±0.06)  20 (83%) | 4 (17%)  (20 ±0.1)  20 (83%) | 0.89  0.49 |
| Abdominal pain in last 10 days | 0.38 ±0.21 | 0.62 ±0.3 | 1.2 ±0.5 | 0.5 ±0.24 | 0.37 |
| Current fullness, bloating, swelling  Yes (%) (severity^2^)  No (%) | 6 (29%)  (26 ±0.01)  15 (71%) | 4 (19%)  (16 ±0.06)  17 (81%) | 7 (29%)  (27 ±0.05)  17 (71%) | 5 (21%)  (24 ±0.05)  19 (79%) | 0.81  0.22 |
| Bowel habit satisfaction^3^ | 45 ± 5.8 | 26 ± 4.3** | 50 ±5.1 | 21 ±0.5*** | 0.24 |
| Interference of bowl habit with life^4^ | 23 ±4.3 | 10 ±3.3** | 33 ±4.5 | 15 ±3.5** | 0.28 |
| Urgency to have bowl movement^4^ | 17 ±4.6 | 6.3 ±2.4* | 17 ±3.5 | 12 ±3.5 | 0.32 |

^1^p-value for post-intervention diet vs. control; within same row and group difference between pre-and post-intervention at *p≤0.05, **p≤0.01, ***p≤0.001

VAS scale:

^2^mild (0-25%), moderate (26-50%), severe (27-75%), very severe (76-100%)

^3^Quite happy (0-33%), unhappy (34-66%) very unhappy (67-100%)

^4^Not much (0-33%), quite a lot (34-66%), completely (67-100%)

**Supplementary Table 4**. Diet intervention did not affect levels of serum CRP and IFN γ, TNF α, IL-12 p70 or IL-6 in unstimulated and LPS-stimulated whole bloods.

| **Inflammatory marker** | **Control** | | **Diet** | | **p-value^1^** |
| --- | --- | --- | --- | --- | --- |
|  | **Pre** | **Post** | **Pre** | **post** |  |
| CRP (mg/L) | 0.82 (1.2) | 1.5 (1.9) | 0.98 (1.02) | 0.64 (1.53) | 0.6 |
| **Cytokines** (pg/mL) |  | |  | |  |
| ***Unstimulated*** | | | | | |
| IL-10 | 0.23 (0.14) | 0.29 (0.15) | 0.27 (0.23) | 0.33 (0.24) | 0.6 |
| IL-8 | 143 (853) | 82 (119) | 135 (485) | 118 (224) | 0.8 |
| INF-γ | 0.63 (1.46) | 0.75 (2.1) | 0.66 (0.88) | 0.64 (0.44) | 0.2 |
| IL-12 p70 | 0.08 (0.24) | 0.13 (0.14) | 0.1 (0.12) | 0.12 (0.15) | 0.8 |
| IL-6 | 0.3 (0.71) | 0.22 (0.18) | 0.22 (0.26) | 0.19 (0.14) | 0.9 |
| TNF-α | 0.56 (1.6) | 0.79 (0.85) | 0.57(0.89) | 0.72 (0.67) | 1.0 |
| ***TLR-4 (LPS) stimulated*** | | | | | |
| IL-10 | 173 (121) | 176 (169) | 236 (152) | 269 (279) | 0.02 |
| IL-8 | 1559 (105) | 1478 (91)* | 1516 (108) | 1432 (108)* | 0.7 |
| INF-γ | 78.7 (85.8) | 48 (129) | 83 (89) | 65 (38) | 0.8 |
| IL-12 p70 | 15 (3.9) | 15 (8.4) | 15 (5.2) | 15 (6.9) | 0.3 |
| IL-6 | 1283 (39) | 1290 (57) | 1279 (30) | 1290 (48) | 0.5 |
| TNF-α | 828 (551) | 926 (285) | 791 (661) | 625 (853) | 0.08 |

^1^p-value CONT vs. DIET post intervention; *within same group p≤0.001

Data expressed as median (IQR); n=18 CONT and n=18 DIET

CRP – human ; TLR – toll-like receptor; LPS – lipopolysaccharide; INF – interferon; IL – interleukin; TNF – tumor necrosis factor

**Supplementary Figure 1**. CONSORT Diagram

Expressed Interest (n=492)

**Enrolment**

Pre-screened (n=290)

Excluded (n=59)

Withdrawal prior to randomization (n=17)

Allocation

Randomized (n=47)

Assessed for Eligibility (n=123)

Analyzed (n=24)

Analysis

Allocated to diet intervention (n=24)

Allocated to control intervention (n=23)

Discontinued intervention (n=2)

Analyzed (n=21)

**Supplementary Figure 2**. Bristol Stool Chart


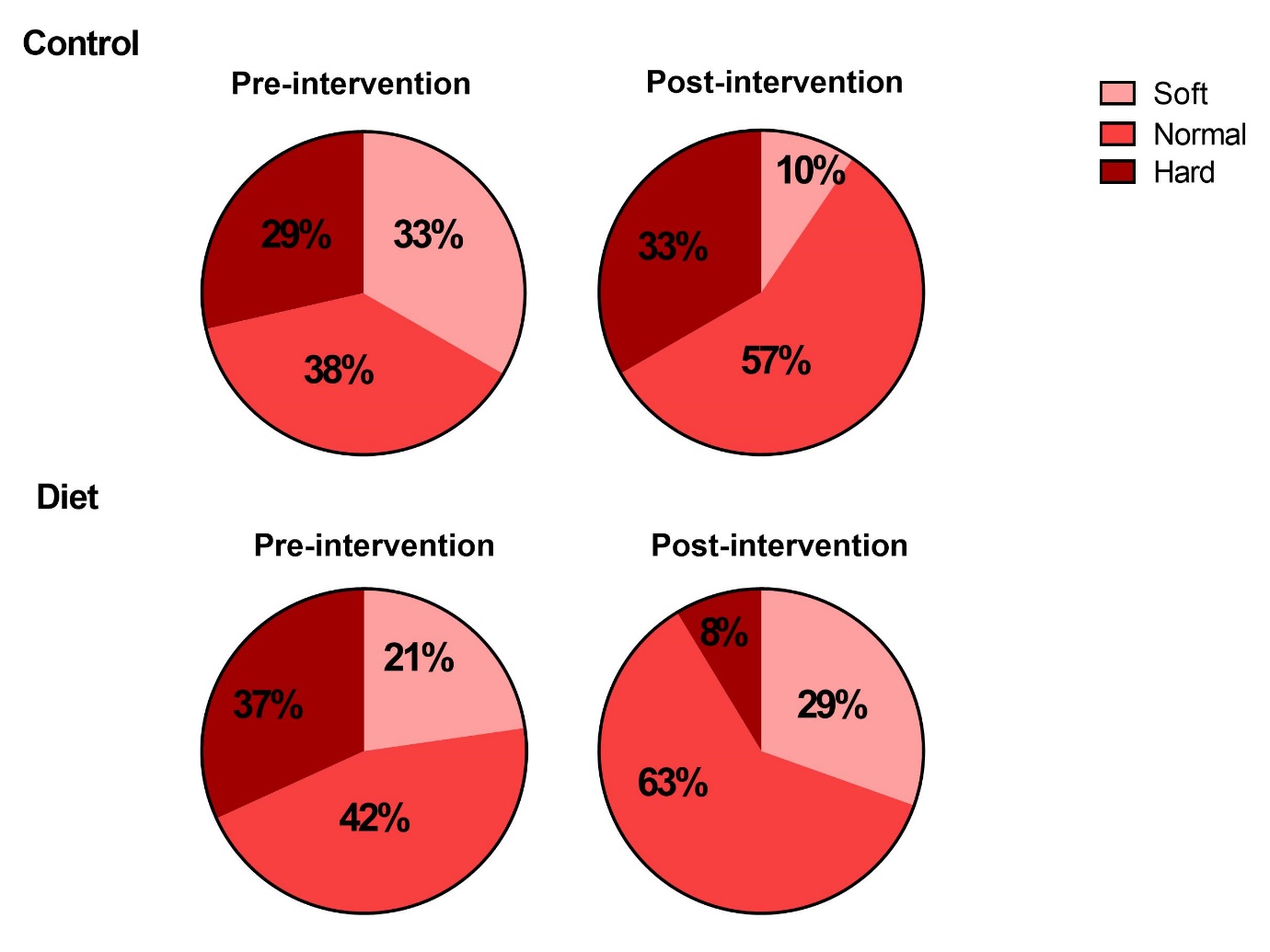


**Supplementary Figure 3**. Impact of psychobiotic and control diet on subjective sleep quality during intervention

1. b)


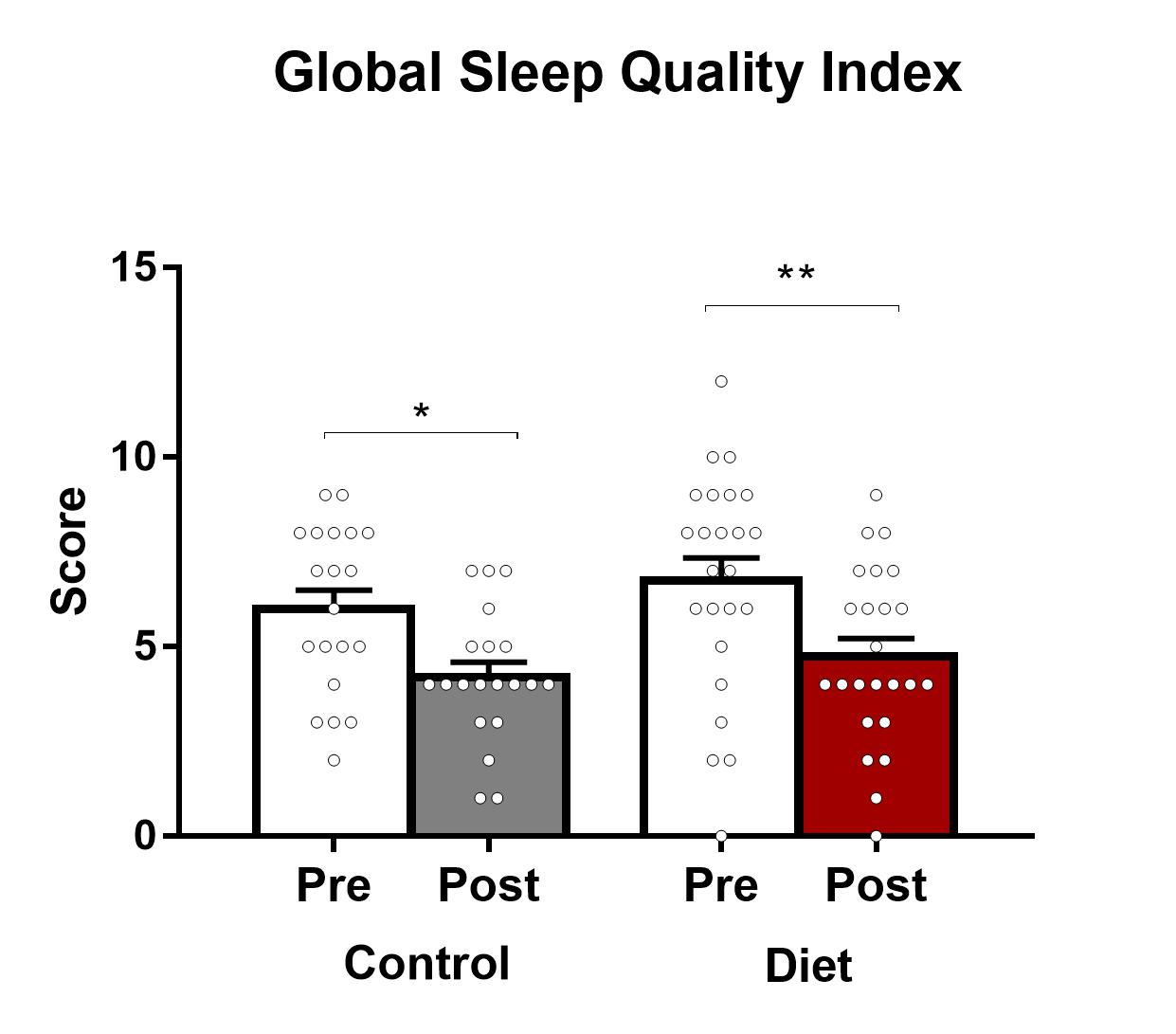


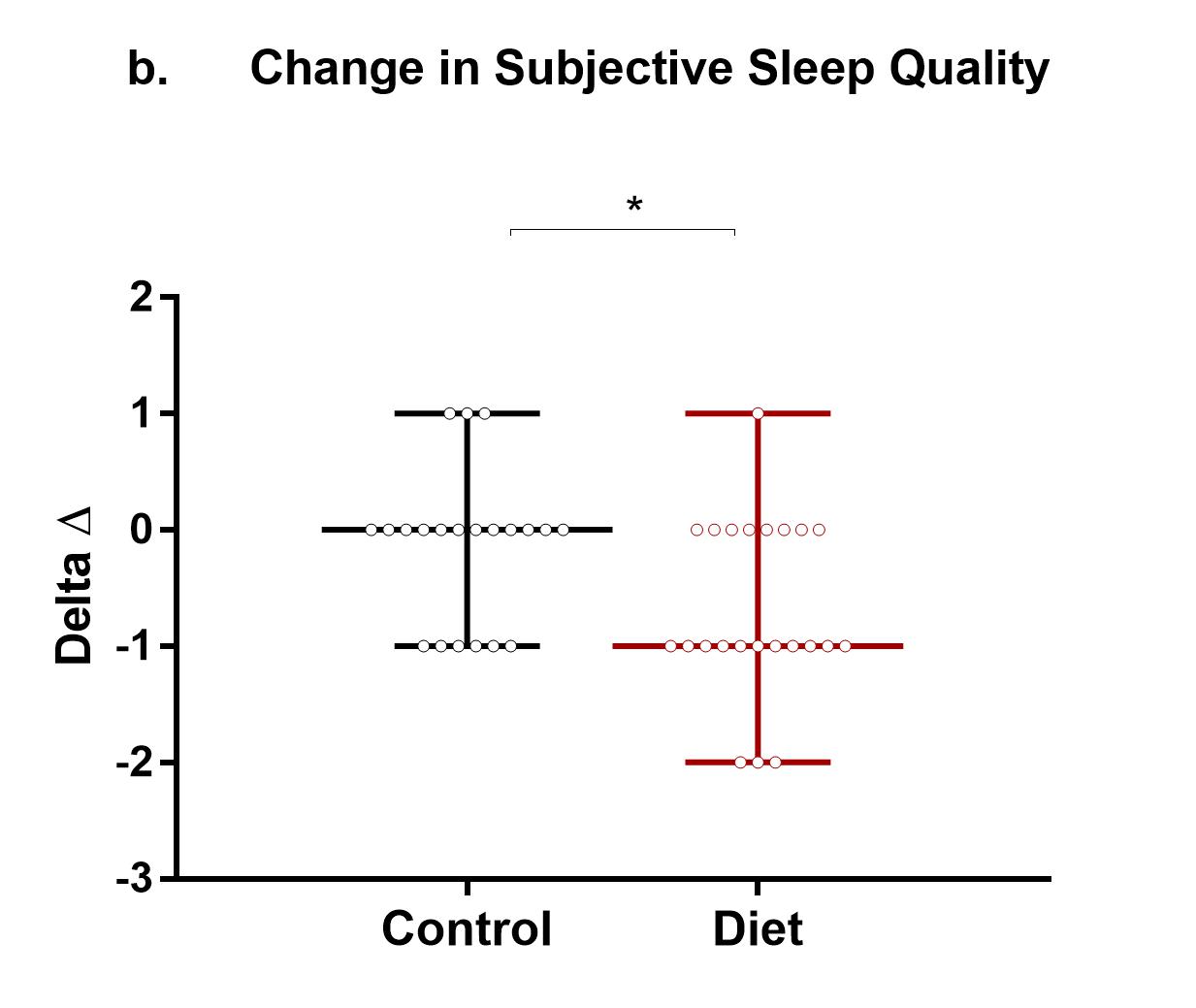


Impact of both intervention arms on a) global sleep quality and b) subjectve sleep quality as measured by Pittsburgh Sleep Quality Index. Global sleep quality expressed as mean±SEM; change in sleep quality component scores expressed as median (IQR); *p≤0.05; p≤0.01

**Supplementary Figure 4**. HPA axis response was not affected by dietary intervention

1. b)


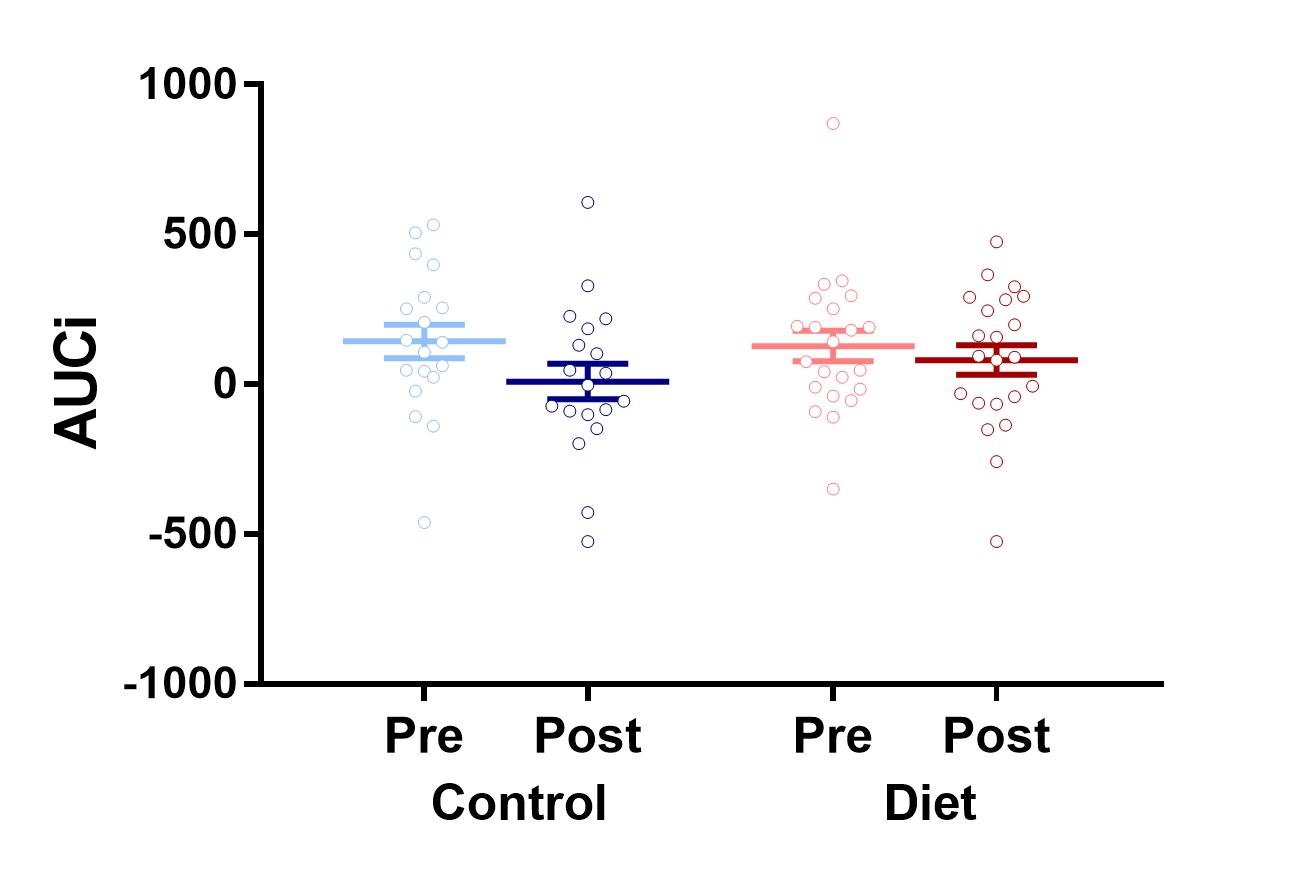


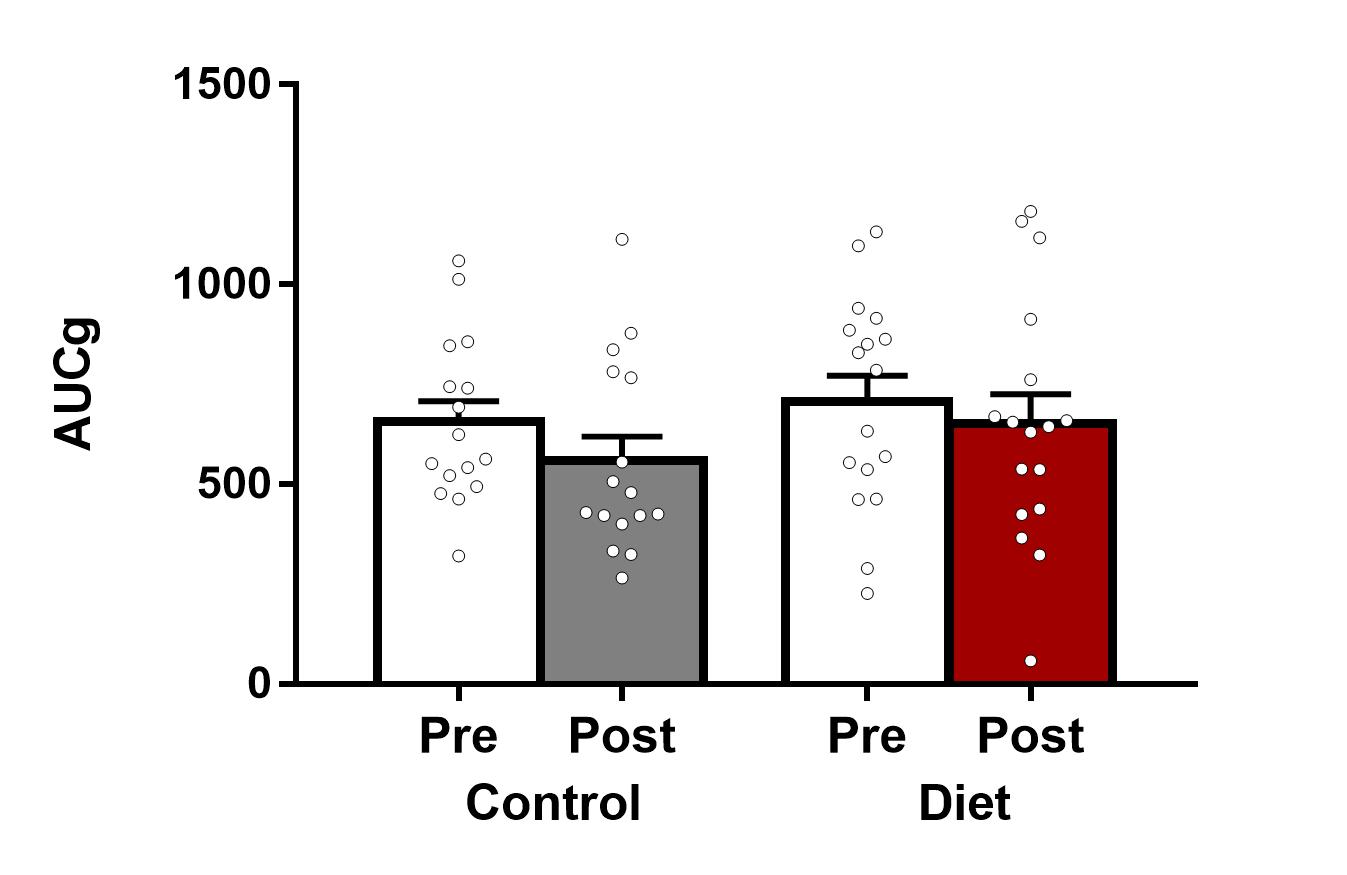


Morning cortisol levels were not significantly affected by dietary intervention. a) CAR levels shown as area under the curve with respect to ground (AUCg) pre- and post intervention in both groups; b) CAR levels shown as area under the curve with respect to increase; CAR expressed as mean ± SEM
