# Supplementary material for: Feed your microbes to deal with stress: a psychobiotic diet impacts microbial stability and perceived stress in a healthy adult population
Source: Mol Psychiatry. 2022 Oct 27;28(2):601–10. doi: 10.1038/s41380-022-01817-y (PMC9908549; doi:10.1038/s41380-022-01817-y)
Supplement: Supplementary file 2 — Diet Education Material [file 41380_2022_1817_MOESM2_ESM.pdf]

Interfacing Food & Medicine

Dear participant,

Below is a list of foods that you will focus on during the 4-week intervention. You can also find different ideas of how to incorporate these foods into your daily diet on the next page.

List of Foods to Include

Fruits (3 servings/day) and Vegetables (6 servings/day)

- Apples
- Bananas
- Berries
- Peaches
- Onions
- Cabbage/Chicory
- Leeks
- Asparagus
- Garlic
- Carrots
- Sweet potatoes
- Bell Peppers
- Artichokes/Jerusalem Artichokes

Grains (5 to 8 servings/day) and Legumes (3 to 4 servings/week)

- Whole grain products
- Oats
- Quinoa
- Barley
- Nuts
- Chia Seeds
- Chickpeas/Hummous
- Lentils
- Flax seeds (ground)
- Beans

Fermented Foods (2-3 servings/day)

- Kefir
- Sauerkraut
- Kombucha
- Probiotic-containing products (i.e., yoghurts, Yakult)

Microbiome Ireland
Interfacing Food & Medicine

How can you use these foods in your diet?

- Aim for eating 6-9 servings of fruits and vegetables per day. Most of these should include the fruits and vegetables listed above.
- Fruit: Eat a whole apple or slice it up and eat with your cereal or oat. It also makes a great addition to smoothies with other fruits. For example, you can add some Kefir and fruits in a blender and make a smoothie rich in prebiotics
- Vegetables: Oven roasted vegetables are easy to make and add an extra crunch. You can also add some fresh garlic which enhances taste and adds additional prebiotics.
- Grains: Consume whole grains instead of refined grains (e.g., white bread).
- Sprinkle Chia seeds or flax seeds on your salad on in yogurt or with your oats. You can also add some berries to increase the flavour.
- Kefir: If the Kefir is too bitter, you can make a smoothie by adding some fruit and spices such as cinnamon or a little bit of cocoa.
- Legumes: Quinoa, barley and chickpeas are great additions to salads. Here you can also add some onions and other vegetables to enhance the flavour and adding more prebiotics.
- You can buy canned chickpeas for easy use. Quinoa and barely have to be cooked before use. However, just like boiling rice, it is very easy to prepare. You can also find prepared food with quinoa which are easier to use.
- Instead of chickpeas, you can also eat hummous. Pair them with crackers that are made of whole wheat or quinoa and other grains.
- Nuts: A handful of nuts make a great snack for in-between meals.
- Kombucha is a fermented black or green tea. You can enjoy it with your meal or as a drink to sip on during the day.
- Sauerkraut can be enjoyed in various different ways. Try adding it to soups, casseroles, sandwiches or simply enjoy it with some mashed potatoes.
- Leeks and cabbage: Prepare a stir fry as a delicious way to enjoy leeks and cabbage. You can also add some onions and garlic for more flavour.

apc Microbiome Ireland
Interfacing Food & Medicine

Example Meal Plan

Meal	Day 1	Day 2	Day 3	Day 4	Day 5	Day 6	Day 7
**Breakfast**	Porridge or oat (2/3 cup) with 1 banana	Kefir smoothie with fruit (from fruit list) and flax seeds (250 mL)<br>1 slice of whole wheat bread with peanut butter	Scrambled eggs on whole wheat toast with avocado and tomatoes	Porridge or oat (1/3 cup) with ½ cup of blueberries	Yoghurt (e.g., Activia) with flax seeds and berries	Whole grain cereal (e.g., Bran flakes) with milk<br>½ cup of berries	Kefir smoothie with fruit and flax seeds (250 mL)<br>1 slice of whole wheat bread with peanut butter
**Morning Snack**	Handful of nuts (with skin)<br>½ cup of berries	1 Orange	1 Yoghurt (e.g., Activia) with fruit	Handful of nuts<br>½ cup of berries	1 banana with peanut butter<br>High fibre granola bar	1 cup of Kefir<br>1 medium apple	2 Mandarins<br>Handful of nuts
**Lunch**	Whole grain bread with crambled eggs with sauerkraut, and avocado**(R)**	Reuben sandwich (Rye bread, corned beef and Sauerkraut) **(R)**	Chickpea Salad Wraps with Sauerkraut and vegetables **(R)**	Barley vegetable soup with chorizo **(R)**	Sandwich (whole grain bread, low fat cheese, lunch meat, lettuce, tomatoes, cucumbers)	Chicken sauerkraut apple Panini **(R)**	Tuna, avocado, quinoa salad **(R)**
**Afternoon Snack**	1 medium apple<br>1 cup of Kefir	1 Yoghurt (e.g., Activia) with fruit (from fruit list)	1 banana<br>1 cup of Kefir	1 medium apple<br>1 cup of Kefir	Hummous with 6 wholemeal crackers and carrots<br>1 cup of Kefir	Handful of nuts<br>2 mandarins	Hummous with 6 wholemeal crackers and carrots
**Dinner**	Grilled chicken, oven roasted asparagus (with olive oil and garlic) and 1 medium sweet potato	Whole grain spaghetti with Chia seed meatballs and marinara sauce **(R)**<br>Green salad	Vegetable stir fry with pork loin and brown rice	Whole grain chicken pasta with vegetables and cabbage and tomato sauce	Beans and cabbage soup with slice of whole grain bread **(R)**	Mediterranean salad and fish **(R)**	Lean ground beef and cabbage casserole **(R)**

*Drink a cup of Kombucha with your lunch or dinner or as a snack during the day

Examples of Food groups and serving sizes

Estimating serving sizes

Use a disposable plastic cup to guide portion sizes of grains and cereal, vegetables and beverages

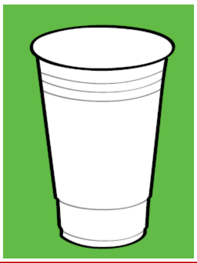

Vegetables

1 serving equals

½ disposable 200 mL cup cooked or raw vegetables, 1 disposable 200 mL cup of leafy vegetables

Fruit

1 serving equals

1 medium piece of fruit (i.e., apple), 2 smaller size fruit (i.e., mandarins), 10 grapes, 6 strawberries, ½ cup blueberries

Grains, Nuts and Legumes

1 serving equals

1 slice of whole wheat bread, 2 breakfast wheat or oat biscuits, 1/3 cup of raw porridge oats, 6 wholemeal crackers, 1 cup sweet potatoes or ½ cup of cooked whole wheat pasta or brown rice
Handful (30g) of nuts, 1-2 teaspoons of peanut butter, 2-3 tablespoons of seeds
½ disposable 200 mL cup of lentils, beans or chickpeas

Fermented foods

1 serving equals

1 cup of kefir or Kombucha, ½ cup of sauerkraut, ¾ cup of yoghurt

Barley-Vegetable Soup with Chorizo

Ingredients:

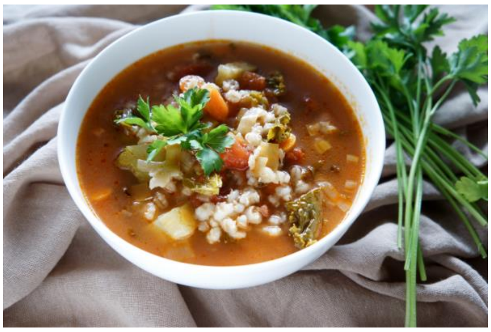

- 1 tbsp olive oil
- 200g Chorizo (sliced and quartered)
- 2 large carrots (chopped)
- 1 leek (chopped)
- 1.5 tbsp tomato paste
- 900 mL vegetable stock
- 1 can diced tomatoes
- 90 g barely (cooked)
- 1 courgette
- 2 handful of kale leaves (chopped)
- Salt, pepper, red pepper flakes and parsley to taste

Preparation:

1. In a pot on medium heat, heat the olive oil and add chorizo. Cook until chorizo is crisp on edges, stirring occasionally.
2. Add carrots and leeks. Cook until leeks are translucent and carrots have softened. Add tomato paste, vegetable stock and canned tomatoes, stirring until everything is fully incorporated.
3. Once the soup beings to simmer, add the remaining ingredients. Simmer for 15 minutes until courgette is cooked and kale is tender. Season to taste.

(Source: http://www.carolyngcreates.com/2016/10/03/barley-soup-with-smoked-chorizo/)

Bean and Cabbage Stew

Ingredients:

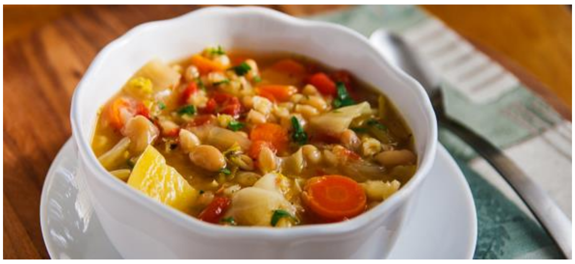

- 1 large onion, chopped
- 3 ribs celery, chopped
- 2 to 3 cloves garlic, minced
- ½ head cabbage, chopped
- 4 carrots, sliced
- 1 to 1.5 lbs potatoes, diced
- 1/3 cup pearled barley
- 1 bay leaf
- 1 tsp thyme
- ½ tsp rosemary
- ½ tsp black pepper
- 1.5 L vegetable broth
- 2 cans beans, drained
- 1 can diced tomatoes
- 1 tbsp parsley, chopped
- Salt to taste

Preparation:

4. Place vegetables, seasonings, barley and broth into large pot. Cover and simmer until vegetables are tender, about 45 minutes.
5. Add remaining ingredients and add more seasoning if necessary.
6. Simmer uncovered for 15 minutes.

(Source: https://www.forksoverknives.com/recipes/irish-white-bean-and-cabbage-stew/#gs.9mgLb2MU)

Chia Meatballs with Marinara Sauce

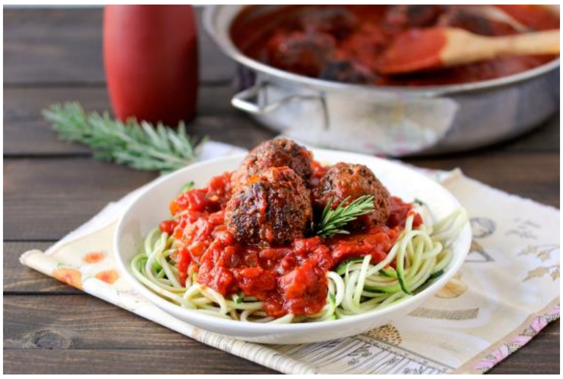

Ingredients:

Chia Meatballs:

- 1 lbs ground beef
- 2 tbsp tomato paste
- 2 cloves garlic (minced)
- 2 tsp Italian herbs
- 1 tsp salt
- 1 tsp pepper
- 2 tbsp chia seeds
- 2 tsp oil (for sautéing; e.g., olive or avocado oil)

Marinara sauce:

- 1 medium onion
- 1 clove garlic
- 125 mL beef or chicken stock
- 1 can diced tomaotes
- 1 can tomato sauce
- Salt and pepper to taste
- Seasoning (e.g., rosemary, oregano)

Preparation:

1. Combine all ingredients (ground beef, tomato paste, garlic, herbs, salt, pepper, chia seeds) in a bowl. Let rest for 10 minutes.
2. Scoop 8 balls and pat into a round shape
3. Heat a large skillet over medium heat and add the oil. Cook meatballs until brown, turning on at least 3 sides. Move meatballs to a place to rest (they will not be fully cooked yet, but will finish cooking later in the sauce.
4. In the same pan, add the onion and sauté until translucent (3-5 minutes). Add garlic and seasoning.
5. Add stock, diced tomatoes, and tomato sauce and cook over medium heat until sauce begins to thicken (15-20 minutes).
6. Reduce heat to medium low and add meatballs. Cook 5-10 minutes or until meatballs are cooked through.
7. Serve with whole grain spaghetti or try spiralized vegetables (such as courgettes).

(Source: https://barerootgirl.com/nourish/2013/9/22/in-season-chia-meatballs-with-easy-marinara-a-giveaway/)

Chicken-Sauerkraut-Apple Panini

Ingredients:

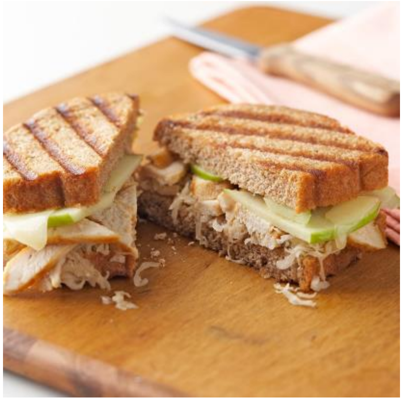

- Sauerkraut
- Whole wheat bread
- Apple
- Chicken breast lunch meat
- Cheese (e.g., Swiss cheese)
- Butter

Preparation:

7. Place sauerkraut in a colander and rinse with cold water. Drain well and press out excess liquid.
8. Coat one side of the bread with butter. Top the other side with chicken, sauerkraut, apple slices and cheese.
9. Preheat a grill pan and add sandwiches. Use a spatula to press the sandwiches down, cooking 6 to 8 minutes on each side or until bottoms are toasted.

(Source: http://www.eatingwell.com/recipe/259852/chicken-sauerkraut-and-apple-panini/)

Chickpea Salad Wrap with Sauerkraut

Ingredients: (3 wraps)

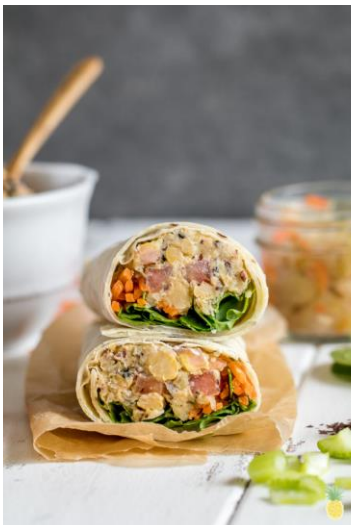

- 1 can chickpeas (drained and rinsed)
- 3 tbsp tahini
- 8 tbsp sauerkraut
- 5 tbsp diced tomatoes
- 4 tbsp diced celery
- 1 tsp rice vinegar
- 1 tbsp lemon juice
- ½ tbsp sesame seeds
- ½ tbsp. dried chives
- Wraps of choice
- Shredded carrots
- Spinach

Preparation:

10. Place chickpeas in a mixing bowl and add the tahini. Mash up the chickpeas with fork or potato masher until almost smooth.
11. Fold in the remaining ingredients and season to taste.
12. Place carrots and spinach on the wrap and top with the chickpea sauerkraut salad. Tip: heat up the wrap in the microwave to make it soft at pliable, if desired.

(Source: https://sweetsimplevegan.com/2017/08/chickpea-sauerkraut-salad-wraps/)

Ground Beef and Cabbage Casserole

Ingredients:

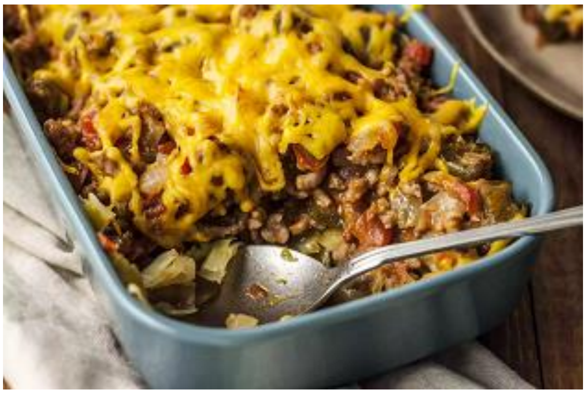

- 1 to 1.5 lbs lean ground beef
- 1 medium onion, chopped
- 1 green bell pepper, chopped
- 1 can diced tomatoes
- 2 tsp cider vinegar
- 1 tsp garlic powder
- 1 head cabbage, coarsely chopped
- 150 g grated Cheddar cheese

Preparation:

13. Preheat oven to 180º C.
14. In a large skillet, cook ground beef, onion and green pepper until meat is no longer pink.
15. Stir in diced tomatoes, vinegar, and garlic powder.
16. Simmer, uncovered, for 10 minutes. Season to taste and set aside.
17. Put about 3 cm of water and a pinch of salt in a large saucepan. Add the cabbage and bring to a boil. Reduce heat to medium low, cover and cook for about 10 minutes. Drain thoroughly.
18. Arrange cabbage in a casserole pan. Pour ground beef mixture over cabbage and bake uncovered for 20-25 minutes.
19. Sprinkle cheese over the casserole and bake for another 5 minutes, until cheese has melted.

(Source: https://www.thespruceeats.com/ground-beef-and-cabbage-casserole-3057387)

Mediterranean Salad

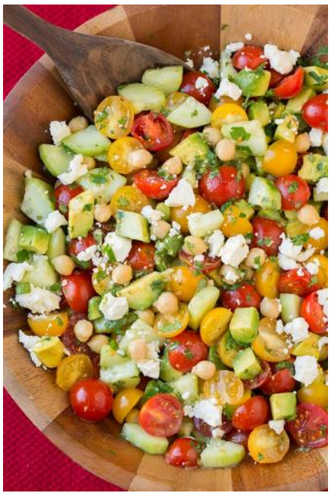

Ingredients:

- 1 container red cherry tomatoes, halved
- 1 container yellow cherry tomatoes, halved
- 1 can chickpeas, drained and rinsed
- 1 cucumber, diced
- 1 avocado, diced
- 1 pack feta cheese, crumbled
- 4 tbsp olive oil
- 2 tbsp lemon juice
- 2 cloves garlic, mined
- Fresh basil

Preparation:

20. In a salad bowl toss together tomatoes, chickpeas and cucumber.
21. Add avocado and feta and toss gently.
22. Add olive oil, lemon juice, garlic and basil and toss until evenly coated.

(Source: https://www.cookingclassy.com/tomato-avocado-cucumber-chick-pea-salad-with-feta-and-greek-lemon-dressing/)

Reuben Sandwich

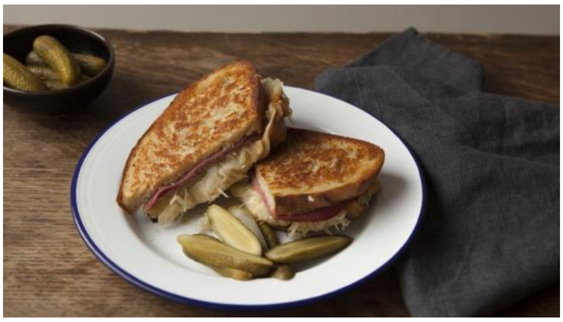

Ingredients:

- Rye or sourdough bread
- Butter
- 2-3 slices corned beef
- 1 slice Swiss cheese (or Emmental or Gruyère)
- 2-3 tbsp sauerkraut
- 1 tbsp Thousand Island Dressing

Preparation:

1. Spread the Thousand Island dressing on both slices of bread. Then put the corned beef on one slice and top it with sauerkraut and the cheese.
2. Heat sandwich in a sandwich maker or frying pan. If using frying pan, put butter in the pan, press sandwich down onto the pan with a spatula and health for 3-4 minutes from each side until the bread is browned and the cheese has melted.

(Source: https://www.bbc.com/food/recipes/reuben_sandwich_25821)

Tuna-Avocado-Quinoa Salad

Ingredients:

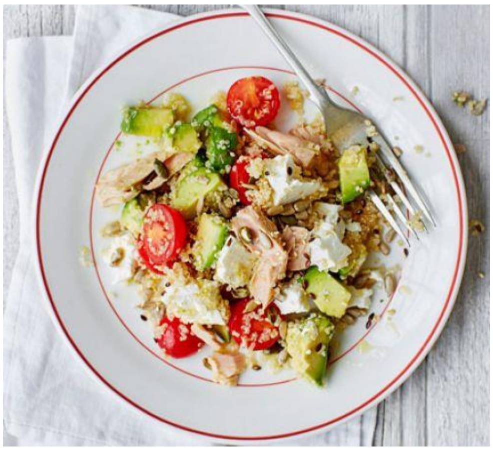

- 100 g quinoa
- 3 tbsp extra virgin olive oil
- Juice from 1 lemon
- ½ tbsp white wine vinegar
- 120 g can tuna, drained
- 1 avocado, diced
- 200g cherry tomatoes, halved
- 50g feta, crumbled
- 50g baby spinach
- 2 tbsp mixed seeds, toasted

Preparation:

23. Rinse quinoa under cold water and prepare according to package instructions. Transfer to a bowl to cool.
24. In another bowl, combine the oil, lemon juice and vinegar with some seasoning.
25. Once quinoa has cooled, mix with the dressing and remaining ingredients.

(Source: https://www.bbcgoodfood.com/recipes/tuna-avocado-quinoa-salad)

Whole grain bread with scrambled eggs, avocado and sauerkraut

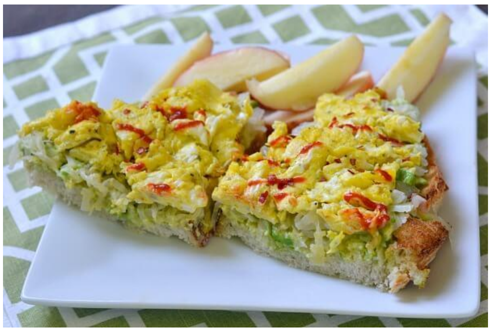

Ingredients:

- Whole grain bread
- Sauerkraut
- Avocado
- Scrambled eggs

Preparation:

3. Cut the avocado in half, scoop it out and spread it on the bread. *Tip*: You can toast the bread to give it some extra crunchiness.
4. Add a layer of sauerkraut. You can use as much or as little as you want.
5. Scramble your eggs. Season with salt and pepper or other seasonings to taste. Then top the sandwich with the scrambled eggs.
6. *Tip*: You can add other vegetables (e.g., tomato) or any sauces (e.g., Siracha sauce) to the sandwich to cater it to your taste.

(Source: https://www.peanutbutterrunner.com/avocado-sauerkraut-and-scrambled-egg-toast/)

Microbiome Ireland
Interfacing Food & Medicine

Weekly Checklist

**Food Group**<br>Ideal serves	**Food Consumption**<br>This checklist will help you to remember what to eat every day.<br>Place a tick (✓) in the box every time you have a single serve from the food group listed.						
	**Day 1**	**Day 2**	**Day 3**	**Day 4**	**Day 5**	**Day 6**	**Day 7**
**Vegetables**<br>6 servings/day	☐☐☐<br>☐☐☐	☐☐☐<br>☐☐☐	☐☐☐<br>☐☐☐	☐☐☐<br>☐☐☐	☐☐☐<br>☐☐☐	☐☐☐<br>☐☐☐	☐☐☐<br>☐☐☐
**Fruit**<br>3 servings/day	☐☐☐	☐☐☐	☐☐☐	☐☐☐	☐☐☐	☐☐☐	☐☐☐
**Grains**<br>5 to 8 servings/day	☐☐☐<br>☐☐☐<br>☐☐	☐☐☐<br>☐☐☐<br>☐☐	☐☐☐<br>☐☐☐<br>☐☐	☐☐☐<br>☐☐☐<br>☐☐	☐☐☐<br>☐☐☐<br>☐☐	☐☐☐<br>☐☐☐<br>☐☐	☐☐☐<br>☐☐☐<br>☐☐
**Fermented Foods**<br>2 per day	☐ ☐	☐ ☐	☐ ☐	☐ ☐	☐☐	☐ ☐	☐ ☐
**Legumes**<br>3 to 4 servings/week	☐ ☐ ☐ ☐						

Healthy Food for Life

www.healthyireland.ie

hi Healthy Ireland

The **Food Pyramid**

For adults, teenagers and children aged five and over

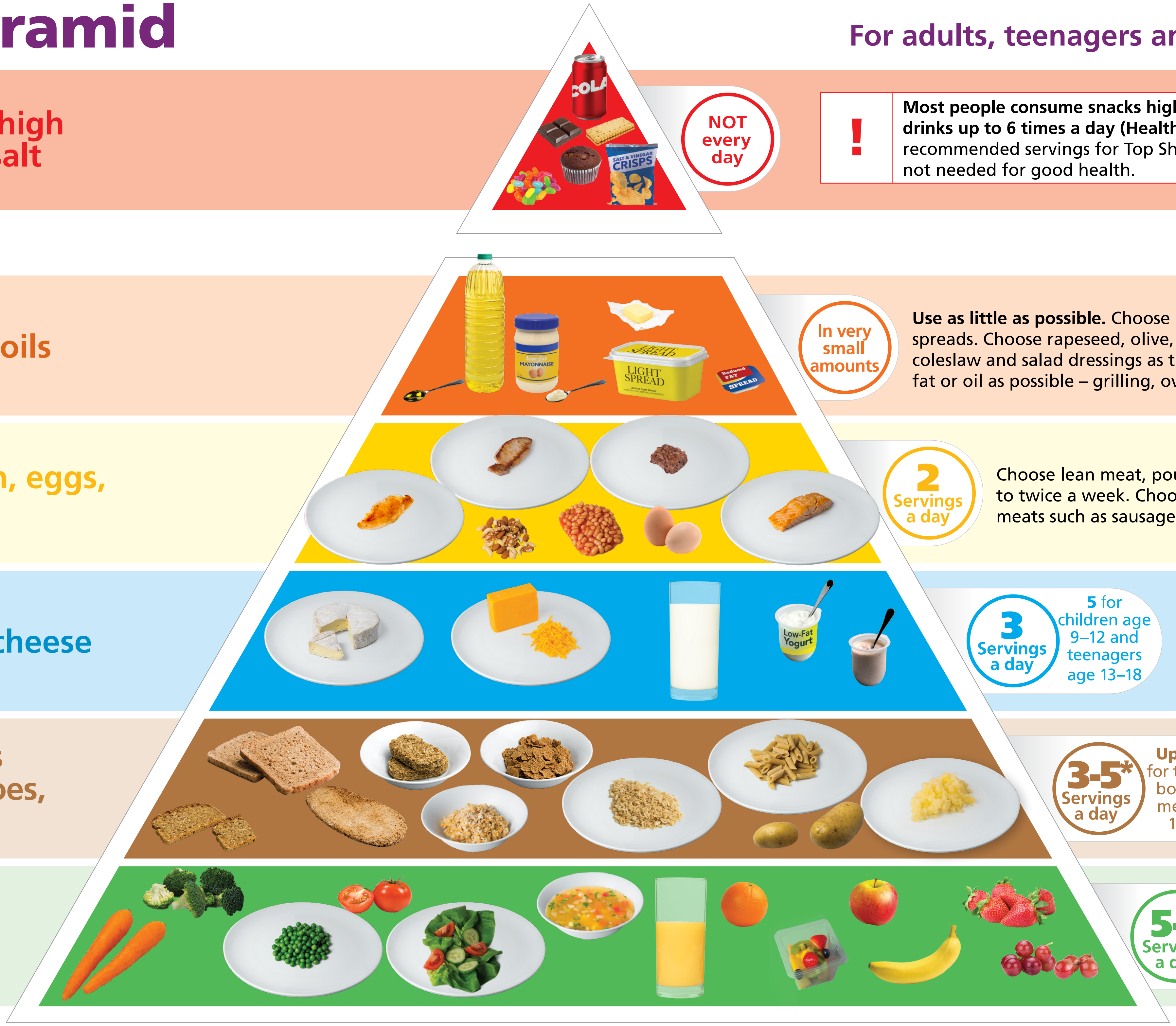

Not needed for good health.

Foods and drinks high in fat, sugar and salt

NOT every day

! **Most people consume snacks high in fat, sugar and salt and sugar sweetened drinks up to 6 times a day (Healthy Ireland Survey 2016).** There are no recommended servings for Top Shelf foods and drinks because they are not needed for good health.

Needed for good health. Enjoy a variety every day.

Fats, spreads and oils

In very small amounts

Use as little as possible. Choose mono or polyunsaturated reduced-fat or light spreads. Choose rapeseed, olive, canola, sunflower or corn oils. Limit mayonnaise, coleslaw and salad dressings as they also contain oil. Always cook with as little fat or oil as possible – grilling, oven-baking, steaming, boiling or stir-frying.

Meat, poultry, fish, eggs, beans and nuts

2 Servings a day

Choose lean meat, poultry (without skin) and fish. Eat oily fish up to twice a week. Choose eggs, beans and nuts. Limit processed salty meats such as sausages, bacon and ham.

Milk, yogurt and cheese

3 Servings a day

5 for children age 9–12 and teenagers age 13–18

Choose reduced-fat or low-fat varieties. Choose low-fat milk and yogurt more often than cheese. Enjoy cheese in small amounts. Women who are pregnant or breastfeeding need 3 servings a day.

Wholemeal cereals and breads, potatoes, pasta and rice

3-5* Servings a day

Up to 7* for teenage boys and men age 19–50

Wholemeal and wholegrain cereals are best. Enjoy at each meal. The number of servings depends on age, size, if you are a man or a woman and on activity levels. Watch your serving size and use the Daily Servings Guide below.*

Vegetables, salad and fruit

5-7 Servings a day

Base your meals on these and enjoy a variety of colours. More is better. Limit fruit juice to unsweetened, once a day.

*Daily Servings Guide – wholemeal cereals and breads, potatoes, pasta and rice

Active	Child (5–12)	Teenager (13–18)	Adult (19–50)	Adult (51+)	Inactive	Teenager (13–18)	Adult (19–50)	Adult (51+)
	3–4	4	4–5	3–4		3	3–4	3
	3–5	5–7	5–7	4–5		4–5	4–6	4

There is no guideline for inactive children as it is essential that all children are active.

Serving size guide

Cereals, cooked rice and pasta, and vegetables, salad and fruit
Use a 200ml disposable plastic cup to guide serving size.

Cheese
Use two thumbs, width and depth to guide serving size.

Meat, poultry, fish
The palm of the hand, width and depth without fingers and thumbs, shows how much you need in a day.

Reduced-fat spread
Portion packs found in cafes can guide the amount you use. One pack should be enough for two slices of bread.

Oils
Use one teaspoon of oil per person when cooking or in salads.

Average daily calorie needs for all foods and drinks for adults

Active 2000kcal **Inactive** 1800kcal

Active 2500kcal **Inactive** 2000kcal

Drink at least 8 cups of fluid a day – water is best

Get Active!
To maintain a healthy weight adults need at least 30 minutes a day of moderate activity on 5 days a week (or 150 minutes a week); children need to be active at a moderate to vigorous level for at least 60 minutes every day.

Source: Department of Health. December 2016.
